# Mechanochemical *Cis/Trans* Isomerization of a Metal Centre Involving a Metal‐Organic Halogen‐Bonded (MOXB) Cocrystal

**DOI:** 10.1002/anie.202517004

**Published:** 2025-10-06

**Authors:** Katarina Lisac, Luzia S. Germann, Mihails Arhangelskis, Martin Etter, Robert E. Dinnebier, Tomislav Friščić, Dominik Cinčić

**Affiliations:** ^1^ Department of Chemistry, Faculty of Science University of Zagreb Horvatovac 102a Zagreb 10000 Croatia; ^2^ Ruđer Bošković Institute Bijenička Cesta 54 Zagreb 10000 Croatia; ^3^ Max Planck Institute for Solid State Research Stuttgart 70569 Germany; ^4^ Department of Chemistry McGill University Montreal H3A 0B8 Canada; ^5^ Faculty of Chemistry University of Warsaw Warsaw 02–093 Poland; ^6^ Deutsches Elektronen‐Synchrotron (DESY) Hamburg 22607 Germany; ^7^ School of Chemistry University of Birmingham Edgbaston Birmingham B15 2TT UK

**Keywords:** Cocrystal, Halogen bond, Isomerization, Mechanochemistry, Metal

## Abstract

Halogen bonding enables the mechanochemical ball‐milling isomerization of an otherwise persistent *cis*‐coordinated metal complex into the corresponding *trans*‐isomer. The importance of halogen bonding for enabling the *cis*→*trans* isomerization of the metal centre is evidenced by real‐time in situ synchrotron powder X‐ray diffraction monitoring of the ball‐milling experiments that showed the transient appearance of a *cis*‐geometry metal‐organic halogen‐bonded (MOXB) cocrystal, which is rapidly replaced by the corresponding *trans*‐geometry one, with any excess, non‐halogen‐bonded *cis*‐geometry complex being retained throughout the milling experiment. The importance of cocrystallization for *cis* **→** *trans* isomerization is supported by periodic density‐functional theory calculations which show that the process becomes notably more enthalpically favourable in the presence of the halogen bond donor. The presented work indicates that the formation of MOXB cocrystals can open the door to new, metal‐based responsive behaviours, different from those of parent solid‐state coordination complexes.

## Graphical abstract

The presented work demonstrates a cocrystallization‐enabled mechanochemical interconversion of geometrical isomers of a coordination complex, illustrating the use of halogen‐bond‐driven cocrystal formation for generating new metal‐based responsive behaviours.

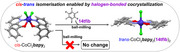

Cocrystallization represents an important supramolecular solid‐state strategy in developing solids with improved or new properties, particularly in the contexts of pharmaceutical solids,^[^ ^1^, ^2^, ^3^ ^]^ agrochemicals,^[^ ^4^, ^5^, ^6^ ^]^ optical materials,^[^ ^7^, ^8^ ^]^ organic semiconductors,^[^ ^9^ ^]^ and energetic materials.^[^ ^10^, ^11^, ^12^ ^]^ The halogen bond has emerged as a highly versatile directional interaction with applications for crystal engineering of multicomponent solids, notably cocrystals.^[^ ^13^, ^14^ ^]^ While the majority of studies on halogen‐bonded cocrystals have focused on organic molecular systems,^[^ ^13^, ^14^, ^15^, ^16^, ^17^ ^]^ much less attention has been dedicated to metal‐organic ones. Metal‐organic halogen‐bonded (MOXB) cocrystals are of interest^[^ ^18^, ^19^, ^20^, ^21^ ^]^ due to the opportunity to introduce metal‐related electrical, magnetic, catalytic, optical and other properties to the self‐assembled materials.^[^ ^22^, ^23^, ^24^ ^]^ Furthermore, metal complexes provide geometries not usually accessible to organic molecules (e.g., square‐planar, trigonal‐bipyramidal, square pyramidal, or octahedral) which makes them desirable as building blocks for new crystal structures. Finally, many metal complexes can form different types of isomers, structural and stereo‐isomers, which expands possibilities for preparing different crystal phases with desirable properties.^[^ ^15^ ^]^ Our group has previously demonstrated a general strategy for the design and synthesis of MOXB cocrystals based on coordination complexes that can engage in halogen bonding via chloride ions coordinated to the metal centre as acceptors.^[^ ^25^, ^26^, ^27^, ^28^ ^]^

Here we report how the formation of a MOXB cocrystal enables mechanochemical *cis*→*trans* isomerization of an octahedral *cis*‐coordinated cobalt(II) complex, which does not undergo such an isomerization on its own. Specifically, whereas solution synthesis selectively provides the crystalline *cis*‐geometry metal complex, which does not undergo isomerization upon mechanical treatment on its own, ball‐milling in the presence of the halogen bond donor 1,4‐diiodotetraflurobenzene (**14tfib**) leads to cocrystallization and subsequent conversion into the corresponding *trans*‐coordinated complex. That the *cis*‐complex on its own does not undergo isomerization and that the mechanochemical *cis*→*trans* isomerization is mediated by the formation of a MOXB cocrystal is evidenced by real‐time in situ synchrotron powder X‐ray diffraction (PXRD) monitoring of milling experiments, as well as by laboratory *ex situ* studies. While previous studies have shown how cocrystallization can stabilize or direct the synthesis of certain isomers of organic molecules,^[^ ^29^, ^30^ ^]^ the current work is to the best of our knowledge the first to demonstrate halogen bond‐based cocrystallization as a means to impart isomerization behaviour to an otherwise inert coordination complex in the solid state.

The focus of this study is the complex *bis*(2‐benzoylpyridine)dichloridocobalt(II) (CoCl_2_ **bzpy**_2_) (Figure 1), obtained as a crystalline solid by solution‐phase reaction of CoCl_2_·6H_2_O and the ligand **bzpy** exclusively in the *cis*‐form (*cis*‐CoCl_2_ **bzpy**_2_), as evidenced by single crystal X‐ray diffraction analysis (see Supporting Information). In contrast, solution synthesis in the presence of **14tfib** was found to lead to three distinct MOXB cocrystals, of compositions (*cis*‐CoCl_2_ **bzpy**_2_)(**14tfib**)_2_, (*cis*‐CoCl_2_ **bzpy**_2_)(**14tfib**), and (*trans*‐CoCl_2_ **bzpy**_2_)(**14tfib**)_2_, sometimes in a mixture, that were all characterized by single crystal X‐ray diffraction (see Supporting Information). The appearance of the (*trans*‐CoCl_2_ **bzpy**_2_)(**14tfib**)_2_ phase is surprising, considering that solution‐based synthesis and crystallization of *cis*‐CoCl_2_ **bzpy**_2_ alone did not lead to the *trans*‐isomer, indicating a role of the XB donor in enabling the *cis*→*trans* isomerization. The crystal structures in all cases reveal one‐dimensional (1D) chains of C─I···Cl─Co halogen bonds involving **14tfib** as the XB donor, with the chloride ligands of the *cis*‐ or the *trans*‐metal‐organic building block acting as the acceptor (Figure 1). In the MOXB cocrystals (*cis*‐CoCl_2_ **bzpy**_2_)(**14tfib**)_2_ and (*trans*‐CoCl_2_ **bzpy**_2_)(**14tfib**)_2_, in which the respective ratio of XB acceptors and donors is 1:2, the 1D chains are further modified by additional **14tfib** molecules: in (*cis*‐CoCl_2_ **bzpy**_2_)(**14tfib**)_2_ the 1D chains are decorated by **14tfib** molecules through I···I halogen bonds, while in (*trans*‐CoCl_2_ **bzpy**_2_)(**14tfib**)_2_ the additional **14tfib** molecules cross‐link the 1D chains through I···Cl halogen bonds to form two‐dimensional (2D) sheets of square‐grid layer (*sql*) topology. The relative thermodynamic stabilities of all cocrystals and reactions leading to their formation were calculated through periodic density‐functional theory (DFT) calculations.^[^ ^31^, ^32^, ^33^ ^]^

**Figure 1:**
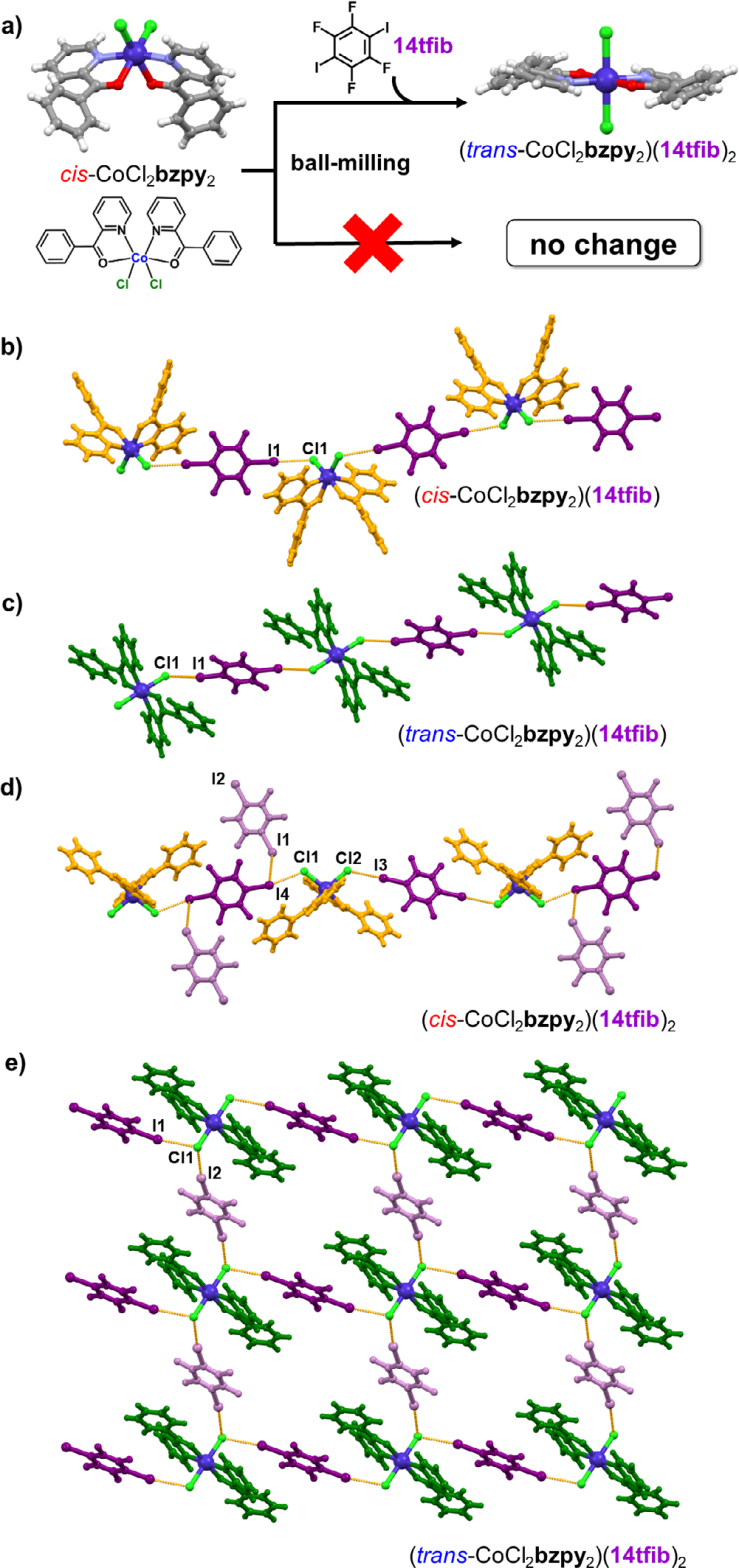
a) The cocrystallization‐enabled *cis*→*trans* isomerization of the complex CoCl_2_ **bzpy**_2_, with the chemical diagram and molecular structures of both isomers shown, as found in pure solid *cis*‐CoCl_2_ **bzpy**_2_ and a **14tfib** cocrystal of *trans*‐CoCl_2_ **bzpy**_2_. Crystal structures of cocrystals: b) (*cis*‐CoCl_2_ **bzpy**_2_)(**14tfib**), c) (*trans*‐CoCl_2_ **bzpy**_2_)(**14tfib**), d) (*cis*‐CoCl_2_ **bzpy**_2_)(**14tfib**)_2_, and e) (*trans*‐CoCl_2_ **bzpy**_2_)(**14tfib**)_2_.The I···Cl halogen bonds are shown as yellow dashed lines. Halogen bonds parameters for b) (*cis*‐CoCl_2_ **bzpy**_2_)(**14tfib**): *d*_I···Cl_ = 3.178(1) Å, ∠_C–I···Cl_ = 177.89(8)°; c) (*trans*‐CoCl_2_ **bzpy**_2_)(**14tfib**): *d*_I···Cl_ = 3.138(5) Å, ∠_C–I···Cl_ = 172.0(2)°; d) (*cis*‐CoCl_2_ **bzpy**_2_)(**14tfib**)_2_: *d*_I3···Cl1_ = 3.189(1) Å, *d*_I4···Cl2_ = 3.197(1) Å; ∠_C–I···Cl_ = 178.9(2)°, ∠_C–I···Cl_ = 167.3(2)°, *d*_I1⋯I4_ = 3.9512(8) Å, ∠_C–I···I_ = 140.29°; e) (*trans*‐CoCl_2_ **bzpy**_2_)(**14tfib**)_2_: *d*_I1···Cl1_ = 3.197(2) Å, *d*_I2···Cl1_ = 3.216(2) Å; ∠_C–I···Cl_ = 166.7(1)°, ∠_C–I···Cl_ = 170.5(1)°.

Next, cocrystal synthesis was attempted mechanochemically, by either ball‐milling in the presence of a liquid additive (liquid‐assisted grinding, LAG)^[^ ^34^, ^35^, ^36^, ^37^, ^38^ ^]^ or by manual grinding with a liquid additive (kneading),^[^ ^39^, ^40^ ^]^ methodologies that both use a small amount of a liquid phase to facilitate transformations (Scheme 1). Kneading reactions were performed by using an agate mortar (60 mm in diameter) and a pestle (18 mm in diameter and 70 mm in length), while LAG reactions were conducted with reaction mixtures placed in stainless steel jars, and shaken at a frequency of 25 Hz using a Retsch MM200 mill using two stainless steel balls of 5 mm diameter (0.5 gram weight each). In all cases, ethanol (EtOH) was used as a liquid additive. The laboratory air temperature during experiments was ca. 25 °C, and relative humidity (RH) varied from 40%–50% (see Supporting Information). The solid reactants and products of mechanochemical screening were characterized by PXRD, differential scanning calorimetry (DSC), and thermogravimetric analysis (TGA). An overview of mechanochemical transformations observed by LAG and kneading is shown in Scheme 1.

**Scheme 1:**
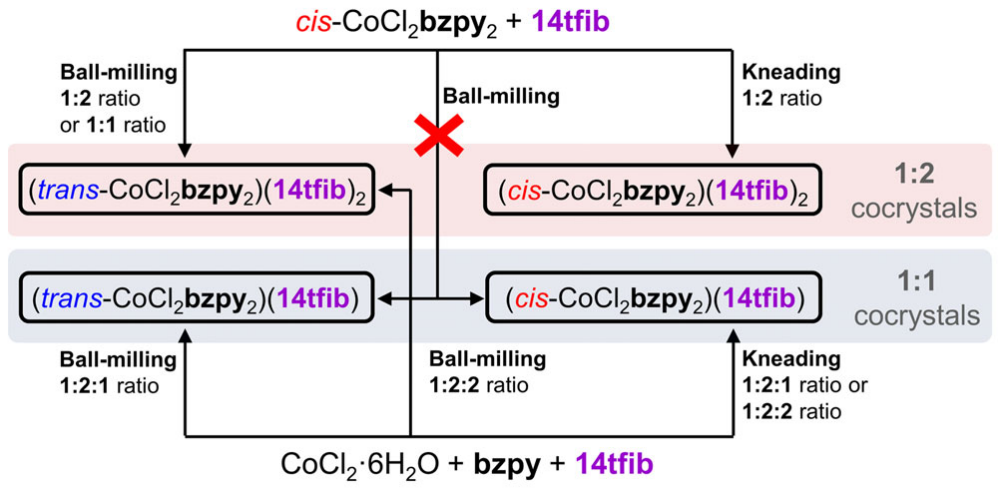
Outcomes of mechanochemical synthesis of the four cocrystals involving CoCl_2_ **Bzpy**_2_ and **14tfib**.

Attempts to synthesize the (*cis*‐CoCl_2_ **bzpy**_2_)(**14tfib**)_2_ cocrystal by 30 min LAG of pre‐synthesized solid *cis*‐CoCl_2_ **bzpy**_2_ and **14tfib** in the respective 1:2 stoichiometric ratio (see Supporting Information) unexpectedly gave the cocrystal of the corresponding *trans*‐isomer (*trans*‐CoCl_2_ **bzpy**_2_)(**14tfib**)_2_, as evidenced by comparison of the PXRD patterns measured for the milled material and calculated for the herein determined crystal structure.^[^ ^41^ ^]^ The pure solid (*cis*‐CoCl_2_ **bzpy**_2_)(**14tfib**)_2_ could not be obtained by ball‐milling, even upon reducing the milling time, frequency, changing the milling assembly (the balls and the jar) from stainless steel to Teflon‐covered,^[^ ^42^ ^]^ or seeding the reaction mixture with pre‐synthesized (*cis*‐CoCl_2_ **bzpy**_2_)(**14tfib**)_2_ (see Supporting Information). To examine whether this outcome could be related to the *cis*→*trans* isomerization of the reagent *cis*‐CoCl_2_ **bzpy**_2_ upon mechanical treatment, the pure solid metal complex was ball‐milled for 1 hour either neat, or in the presence of a small amount of EtOH. Analysis of the ball‐milled material by PXRD in both cases revealed no sign of isomerization (see Supporting Information), indicating that *cis*‐CoCl_2_ **bzpy**_2_ alone does not undergo *cis*→*trans* isomerization by LAG, but requires the presence of **14tfib**. Switching to kneading, however, enabled the synthesis of (*cis*‐CoCl_2_ **bzpy**_2_)(**14tfib**)_2_, indicating that the intensity of mechanical action also plays a role in the *cis*→*trans* isomerization that happens in the presence of **14tfib**.

Next, we explored the LAG reaction of equimolar amounts of *cis*‐CoCl_2_ **bzpy**_2_ and **14tfib** in a ball mill, in expectation to form the MOXB cocrystal containing the XB donors and acceptors in a 1:1 ratio, (*cis*‐CoCl_2_ **bzpy**_2_)(**14tfib**) (Figure 1). However, analysis of the reaction mixture after 30 min revealed the appearance of (*cis*‐CoCl_2_ **bzpy**_2_)(**14tfib**)_2_, with longer milling yielding again (*trans*‐CoCl_2_ **bzpy**_2_)(**14tfib**)_2_, in each case along with residual *cis*‐CoCl_2_ **bzpy**_2_.

To further understand the unexpected *cis*→*trans* isomerization of the *cis*‐CoCl_2_ **bzpy**_2_ moiety upon ball‐milling in the presence of **14tfib**, the mechanochemical reaction was monitored by in situ synchrotron PXRD at the Powder Diffraction and Total Scattering beamline P02.1 of the Deutsches Elektronen‐Synchrotron (DESY). For these real‐time monitoring experiments, the multicomponent reaction mixtures (300 mg of reactants solids, in the presence of 10 µL or 20 µL EtOH) were placed into X‐ray transparent poly(methyl methacrylate) (PMMA) jars, along with two stainless steel balls (7 mm diameter, ca. 1.39 grams each), and the mixtures were milled on a modified Retsch MM400 vibration mill operating at 25 Hz (see Supporting Information). The monitoring experiments were performed on the mechanochemical reactions of *cis*‐CoCl_2_ **bzpy**_2_ and **14tfib** in respective 1:1 and 1:2 stoichiometric ratios, and sequential Rietveld refinement was performed for each in situ experiment. Analysis of the in situ monitoring data for the mechanochemical reaction of 1:1 amounts of *cis*‐CoCl_2_ **bzpy**_2_ and **14tfib** (Figure 2) revealed the appearance of the elusive (*cis*‐CoCl_2_ **bzpy**_2_)(**14tfib**)_2_ after ca. 3 min milling, reaching a maximum content of ca. 45% by weight after ca. 8.5 min. The initially formed (*cis*‐CoCl_2_ **bzpy**_2_)(**14tfib**)_2_ is subsequently replaced by (*trans*‐CoCl_2_ **bzpy**_2_)(**14tfib**)_2_. Notably, the in situ analysis shows that crystalline (*cis*‐CoCl_2_ **bzpy**_2_)(**14tfib**)_2_ almost completely disappears after ca. 15 min, at which point the amount of (*trans*‐CoCl_2_ **bzpy**_2_)(**14tfib**)_2_ remains constant. At the same time, the amount of residual solid *cis*‐CoCl_2_ **bzpy**_2_ also remains constant after the disappearance of (*cis*‐CoCl_2_ **bzpy**_2_)(**14tfib**)_2_, indicating that the mechanochemical *cis*→*trans* isomerization of the metal complex involves the transient formation of the MOXB cocrystal.

**Figure 2:**
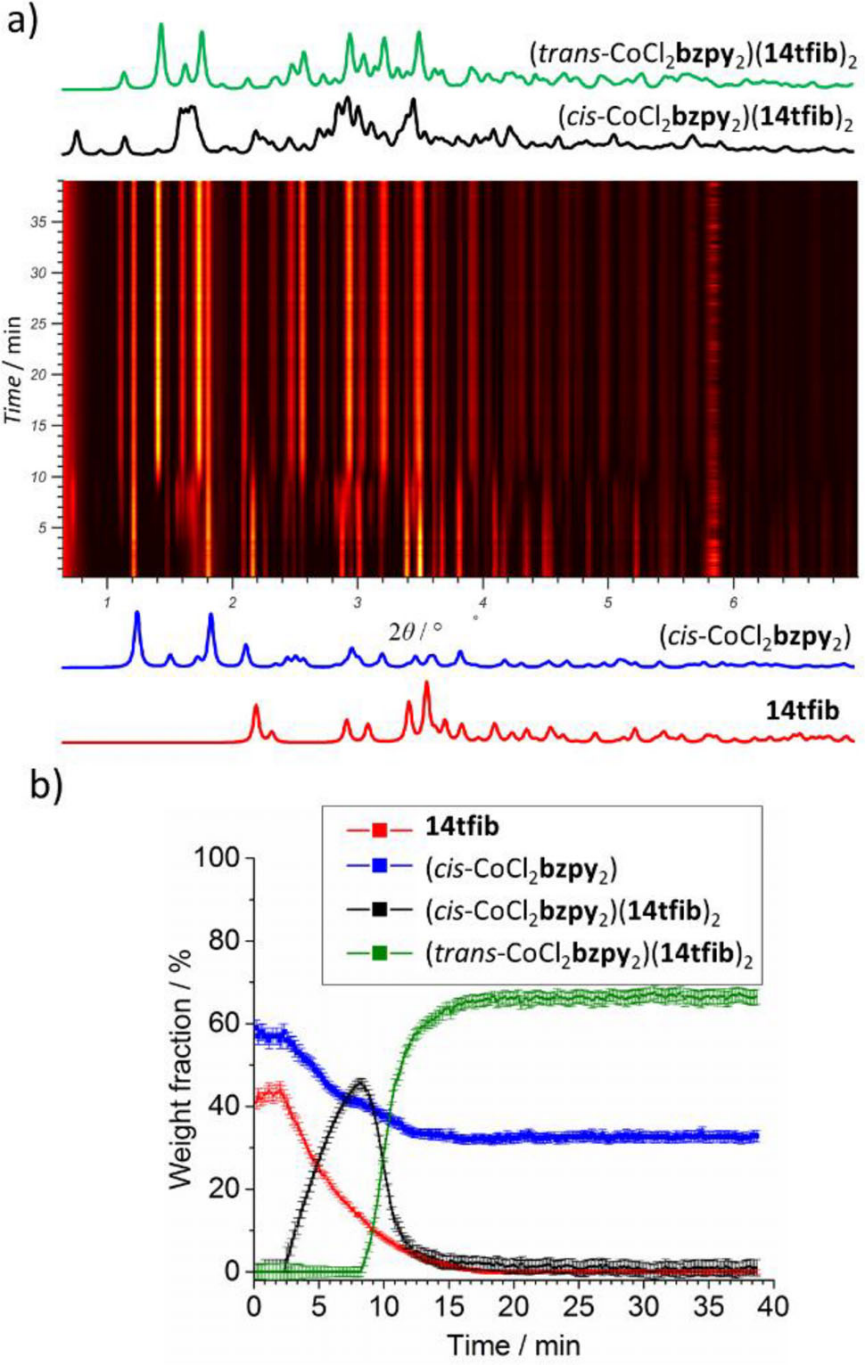
Results of in situ synchrotron PXRD (*λ* = 0.20 709 Å) monitoring of the reaction of equimolar amounts of *cis*‐CoCl_2_ **bzpy**_2_ and **14tfib**: a) the time‐resolved 2D PXRD plot, b) results of quantitative Rietveld analysis of the in situ obtained data.

Similar behaviour was observed for the mechanochemical reaction of *cis*‐CoCl_2_ **bzpy**_2_ and **14tfib** in the respective 1:2 stoichiometric ratio (Figure 3). Real‐time monitoring revealed the rapid formation of (*cis*‐CoCl_2_ **bzpy**_2_)(**14tfib**)_2_, with negligible amounts of residual *cis*‐CoCl_2_ **bzpy**_2_, reaching a maximum abundancy of ca. 56% by weight after ∼2.5 min milling. The subsequent transformation into (*trans*‐CoCl_2_ **bzpy**_2_)(**14tfib**)_2_ reached completion within ca. 7 min milling.

**Figure 3:**
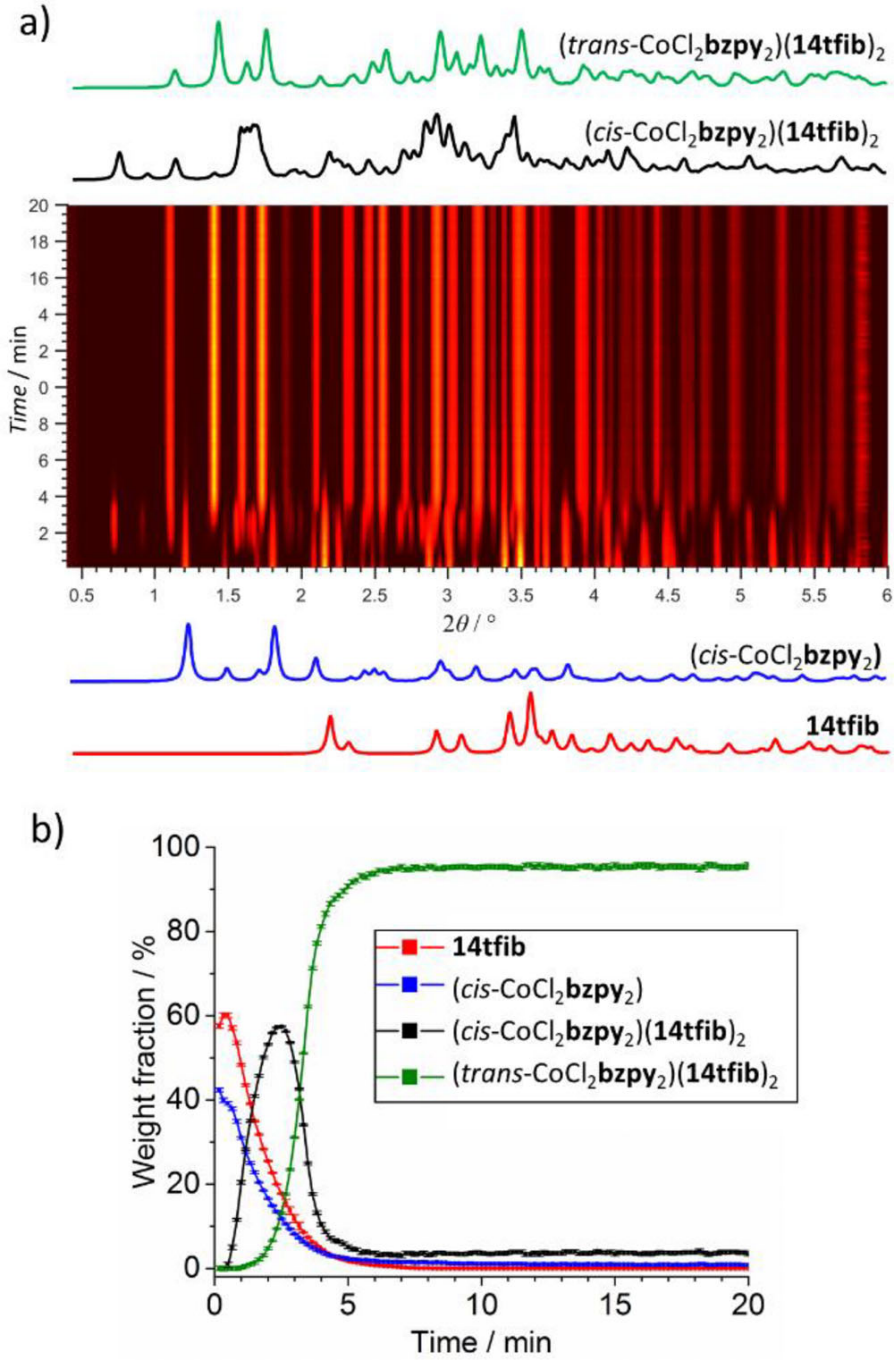
Results of in situ synchrotron PXRD (*λ* = 0.20 709 Å) monitoring of the reaction of *cis*‐CoCl_2_ **bzpy**_2_ and **14tfib** in respective stoichiometric ratio 1:2: a) the time‐resolved 2D PXRD plot, b) results of quantitative Rietveld analysis of the in situ obtained data.

Overall, the outcomes of real‐time and in situ monitoring of mechanochemical cocrystallization of *cis*‐CoCl_2_ **bzpy**_2_ and **14tfib** indicate that, whereas the *cis*‐based MOXB cocrystal readily forms early in the reaction, upon milling it is replaced by the MOXB cocrystal of the corresponding *trans*‐isomer of the metal‐organic unit. Importantly, any residual *cis*‐CoCl_2_ **bzpy**_2_ that does not form the MOXB cocrystal does not undergo isomerization upon continued milling, as evidenced by in situ, as well as *ex situ* analyses.

The so far outlined mechanochemical procedures gave rise to either (*cis*‐CoCl_2_ **bzpy**_2_)(**14tfib**)_2_ or (*trans*‐CoCl_2_ **bzpy**_2_)(**14tfib**)_2_. In contrast, solution one‐pot reactions of CoCl_2_·6H_2_O, **bzpy** and **14tfib** have been observed to produce also the cocrystal containing the XB donor and acceptor in 1:1 stoichiometric ratio, (*cis*‐CoCl_2_ **bzpy**_2_)(**14tfib**). Consequently, the mechanochemical synthesis of (*cis*‐CoCl_2_ **bzpy**_2_)(**14tfib**) was attempted using the multi‐component one‐pot approach. Ball‐milling of CoCl_2_·6H_2_O, **bzpy** and **14tfib** in the respective 1:2:1 stoichiometric ratio along with small amount of EtOH gave after 30 min a new crystalline phase, whose PXRD pattern did not match to any of the starting materials, or the MOXBs obtained from solution. Crystal structure analysis from PXRD data revealed that the new phase is the MOXB cocrystal (*trans*‐CoCl_2_ **bzpy**_2_)(**14tfib**). The corresponding *cis*‐cocrystal, (*cis*‐CoCl_2_ **bzpy**_2_)(**14tfib**), was subsequently obtained by kneading of the starting materials in the presence of EtOH (Scheme 1). Next, the one‐pot multi‐component reactions were explored using CoCl_2_·6H_2_O, **bzpy** and **14tfib** in the respective 1:2:2 stoichiometric ratio. The LAG reaction in presence of EtOH yielded (*trans*‐CoCl_2_ **bzpy**_2_)(**14tfib**)_2_, while kneading gave again the cocrystal (*cis*‐CoCl_2_ **bzpy**_2_)(**14tfib**) along with excess of **14tfib**. Overall, these experiments indicate that the herein employed ball‐milling conditions facilitate the *cis*→*trans* isomerization, leading to the MOXB cocrystal with the *trans*‐CoCl_2_ **bzpy**_2_ core, while kneading enables the cocrystallization with the retention of the *cis*‐isomer structure.

In order to explore whether the isomerization of the *cis*‐CoCl_2_ **bzpy**_2_ unit in the corresponding MOXB cocrystal could be only thermally‐driven, the complex, and all herein prepared cocrystals, were also explored by simultaneous thermogravimetric analysis and differential scanning calorimetry (TGA/DSC). Thermal analysis of solid *cis*‐CoCl_2_ **bzpy**_2_ revealed a sharp endothermic signal with onset around 183 °C, simultaneous with a mass loss, indicating decomposition. The thermograms of the (*cis*‐CoCl_2_ **bzpy**_2_)(**14tfib**)_2_ and the (*trans*‐CoCl_2_ **bzpy**_2_)(**14tfib**)_2_ cocrystals were mutually similar, exhibiting a sharp endothermic signal with an onset around 147 and 137 °C, respectively, in both cases simultaneous with a weight change consistent with the loss of two molecules of **14tfib**. The cocrystals (*cis*‐CoCl_2_ **bzpy**_2_)(**14tfib**) and (*trans*‐CoCl_2_ **bzpy**_2_)(**14tfib**) also exhibited similar thermal signatures, with a sharp endothermic signal around 149 and 150 °C, respectively, in both cases associated with a change in weight indicative of the loss of one equivalent of **14tfib**. Overall, these experiments suggest that none of the MOXB cocrystals, or the solid *cis*‐CoCl_2_ **bzpy**_2_ undergo isomerization before thermal decomposition at high temperatures. Therefore, the observed conversion of (*cis*‐CoCl_2_ **bzpy**_2_)(**14tfib**)_2_ into (*trans*‐CoCl_2_ **bzpy**_2_)(**14tfib**)_2_ appears to be the result of the mechanical LAG treatment.

Finally, the reactions leading to the formation of herein reported MOXB cocrystals and their interconversion were also explored through periodic and molecular density‐functional theory (DFT) calculations. The molecular DFT modelling for isolated *cis*‐ and *trans*‐CoCl_2_ **bzpy**_2_ molecules, performed in Gaussian 16 at PBE/6–311G(d,p)^[^ ^43^, ^44^ ^]^ level of theory, revealed that for a high‐spin state the *cis*‐isomer should be 8.2 kJ mol^−1^ enthalpically more favorable, whereas for a low‐spin state the *trans*‐isomer would be preferred by 12.7 kJ mol^−1^. The preference for the formation of the *trans*‐geometry metal complex is, however, greatly enhanced by cocrystallization: plane‐wave periodic DFT calculations in CASTEP,^[^ ^45^ ^]^ performed with PBE functional combined with many‐body dispersion (MBD*)^[^ ^46^, ^47^, ^48^ ^]^ correction scheme (Table 1), indicated that for the herein observed MOXB cocrystals the *trans*‐isomers should be enthalpically preferred by 70–90 kJ mol^−1^.^[^ ^31^, ^32^, ^33^ ^]^ This result is consistent with observed LAG transformation of (*cis*‐CoCl_2_ **bzpy**_2_)(**14tfib**)_2_ into (*trans*‐CoCl_2_ **bzpy**_2_)(**14tfib**)_2_, which should be enthalpically favoured by ca. 81 kJ mol^−1^.

**Table 1:** Calculated Δ_r_ *H* values (in kJ mol^−1^, per mol of product) for the herein explored reactions of formation and interconversion of MOXB cocrystals based on *cis*‐ and *trans*‐forms of the metal‐organic unit.

Reaction	Δ_r_ *H*/kJ mol^−1^
*cis*‐CoCl_2_ **bzpy**_2_ + 2 **14tfib** → (*cis*‐CoCl_2_ **bzpy**_2_)(**14tfib**)_2_	−9.33
*cis*‐CoCl_2_ **bzpy**_2_ + 2 **14tfib** → (*trans*‐CoCl_2_ **bzpy**_2_)(**14tfib**)_2_	−90.07
(*cis*‐CoCl_2_ **bzpy**_2_)(**14tfib**)_2_ → (*trans*‐CoCl_2_ **bzpy**_2_)(**14tfib**)_2_	−80.74
*cis*‐CoCl_2_ **bzpy**_2 _+ **14tfib** →(*cis*‐CoCl_2_ **bzpy**_2_)(**14tfib**)	−3.36
(*cis*‐CoCl_2_ **bzpy**_2_)(**14tfib**)→ (*trans*‐CoCl_2_ **bzpy**_2_)(**14tfib**)	−70.08

The results of periodic DFT calculations indicate that the formation of the MOXB cocrystal provides an enhanced enthalpic driving force for the herein observed *cis*→*trans* isomerization of CoCl_2_ **bzpy**_2_. A detailed mechanism for this process, however, remains unclear. Tentatively, we suggest that the formation of a halogen bond directly to one of the ligand atoms of the metal complex should weaken the associated coordination bond, overall making the coordination complex more labile, facilitating isomerization to *trans*‐CoCl_2_ **bzpy**_2_ that then leads to the thermodynamically more stable cocrystal. In such a scenario, MOXB cocrystal formation provides the thermodynamic driving force for *cis*→*trans* isomerization, with the formation of individual halogen bonds also making the metal complex sufficiently labile for such a process.

In summary, we have reported that halogen‐bonded cocrystallization enables ball‐milling isomerization of a *cis*‐geometry coordination complex which is otherwise persistent in the solid state. Specifically, whereas solution‐phase synthesis of herein explored CoCl_2_ **bzpy**_2_ complex consistently yields the *cis*‐isomer only, which does not undergo isomerization upon ball‐milling neat or in the presence of a liquid, the formation of a halogen‐bonded metal‐organic (MOXB) cocrystal either from a metal salt or the pre‐synthesized *cis*‐CoCl_2_ **bzpy**_2_ readily leads to *cis*‐*trans* isomerization to form the corresponding *trans*‐isomer as a halogen‐bonded cocrystal. The observed *cis*→*trans* isomerization is also enthalpically‐favored, with the *trans*‐MOXB cocrystal being ca. 70–80 kJ mol^−1^ more exothermic compared to the *cis*‐analogue. The necessity of forming a halogen‐bonded cocrystal prior to ball‐milling isomerization, as well as the persistence of pure solid *cis*‐complex to such isomerization, are supported by real‐time in situ PXRD monitoring of the process, which reveals the initial formation of a halogen‐bonded cocrystal of the *cis*‐complex which is rapidly replaced with the cocrystal of the *trans*‐isomer, whereas any excess solid *cis*‐complex persists throughout the milling experiment. In the context of mechanochemistry, these observations suggest a role for MOXB cocrystals as intermediates in otherwise not accessible mechanochemical transformations of coordination complexes, creating a link to cocrystal‐mediated covalent bonds transformations seen in organic mechanosynthesis.^[^ ^49^, ^50^, ^51^ ^]^ In the broader context of materials and supramolecular chemistry, these results provide a proof‐of‐principle for halogen bond‐driven cocrystal formation as a means to modify reactivity of metal complexes, and present MOXB cocrystals as a class of materials that can exhibit new, responsive behaviours different from those of parent coordination compounds. Further mechanistic studies, as well as systematic exploration of such behavior in other MOXB cocrystal systems, based on diverse metal‐organic complexes and halogen bond donors, are underway.

## Supporting Information

The authors have cited additional references within the Supporting Information.^[^ ^52^, ^53^, ^54^, ^55^, ^56^, ^57^, ^58^, ^59^, ^60^, ^61^ ^]^ Deposition Number(s) 2081192 (for (*trans*‐CoCl_2_ **bzpy**_2_)(**14tfib**)), 2279597 (for (*trans*‐CoCl_2_ **bzpy**_2_)(**14tfib**)_2_), 2279598 (for (*cis*‐CoCl_2_ **bzpy**_2_)(**14tfib**)), 2279599 (for (*cis*‐CoCl_2_ **bzpy**_2_)(**14tfib**)_2_), and 2279600 (for *cis*‐CoCl_2_ **bzpy**_2_) contain(s) the supplementary crystallographic data for this paper. These data are provided free of charge by the joint Cambridge Crystallographic Data Centre and Fachinformationszentrum Karlsruhe Structures service.

## Conflict of Interests

The authors declare no conflict of interest.

## Supporting information



Supporting Information

Supporting Information

## Data Availability

The data that support the findings of this study are available in the Supporting Information of this article.
