# Supplementary material for: Mechanochemical Cis/Trans Isomerization of a Metal Centre Involving a Metal‐Organic Halogen‐Bonded (MOXB) Cocrystal
Source: Angew Chem Int Ed Engl. 2025 Oct 6;64(47):e202517004. doi: 10.1002/anie.202517004 (PMC12624328; doi:10.1002/anie.202517004)
Supplement: Supplementary file 1 — Supporting Information [file ANIE-64-e202517004-s002.pdf]

Electronic Supplementary Information

Mechanochemical *cis*/*trans* Isomerization of a Metal Centre Involving a Metal-Organic Halogen-Bonded (MOXB) Cocrystal

Katarina Lisac, Luzia S. Germann, Mihails Arhangelskis, Martin Etter, Robert E. Dinnebier, Tomislav Friščić* and Dominik Cinčić*

Email: dominik@chem.pmf.hr; t.friscic@bham.ac.uk
Tel: +385 1 4606 362

Table of Contents

1. Mechanochemical preparation of pure phases of *cis*-$CoCl_2$**bzpy**$_2$ and cocrystals 2
2. Additional mechanochemical experiments 3
3. Powder X-ray diffraction experiments 4
4. Crystallization 7
5. Crystal structure determination 9
6. *In situ* PXRD monitoring 16
7. Thermal analysis 17
8. Theoretical calculations 19

1. Mechanochemical preparation of pure phases of *cis*-$CoCl_2$bzpy$_2$ and cocrystals

Pure *cis*-$CoCl_2$**bzpy**$_2$ was prepared by ball-milling of $CoCl_2\cdot6H_2O$ (250.0 mg, 1.05 mmol) and **bzpy** (385.0 mg, 2.10 mmol) in a 15 mL stainless steel jar along with 50.0 μL of acetonitrile, and two stainless steel balls 7 mm in diameter and 1.38 g mass for 40 minutes in a Retsch MM200 shaker mill operating at 25 Hz frequency.

The MOXB cocrystal (*cis*-$CoCl_2$**bzpy**$_2$)(**14tfib**)$_2$ was prepared by kneading of the coordination complex *cis*-$CoCl_2$**bzpy**$_2$ (38.2 mg, 0.08 mmol) with halogen bond donor **14tfib** (61.9 mg, 0.016 mmol) in a mortar for 15 min alongside with gradually adding 160 μL of ethanol.

The MOXB cocrystal (*trans*-$CoCl_2$**bzpy**$_2$)(**14tfib**)$_2$ was prepared by LAG of the metal complex *cis*-$CoCl_2$**bzpy**$_2$ (38.2 mg, 0.08 mmol) with halogen bond donor **14tfib** (61.9 mg, 0.15 mmol) in a 5 mL stainless steel jar along with 20.0 μL of ethanol, and two stainless steel balls 5 mm in diameter and 0.50 g mass for 30 minutes in a Retsch MM200 shaker mill operating at 25 Hz or, alternatively, by one-pot LAG reaction of $CoCl_2\cdot6H_2O$ (16.9 mg, 0.10 mmol), **bzpy** (26.0 mg, 0.20 mmol) and halogen bond donor **14tfib** (57.1 mg, 0.20 mmol) in a 5 mL stainless steel jar along with 20.0 μL of ethanol, and two stainless steel balls 5 mm in diameter and 0.50 g mass for 60 minutes in a Retsch MM200 shaker mill operating at 25 Hz.

The MOXB cocrystal (*cis*-$CoCl_2$**bzpy**$_2$)(**14tfib**) was prepared by kneading of $CoCl_2\cdot6H_2O$ (23.6 mg, 0.10 mmol), **bzpy** (36.4 mg, 0.20 mmol) and halogen bond donor **14tfib** (39.9 mg, 0.10 mmol) in mortar for 10 min alongside with gradually adding 40 μL of ethanol.

The MOXB cocrystal (*trans*-$CoCl_2$**bzpy**$_2$)(**14tfib**) was prepared by one-pot LAG of $CoCl_2\cdot6H_2O$ (23.6 mg, 0.10 mmol), **bzpy** (36.4 mg, 0.20 mmol) and halogen bond donor **14tfib** (39.9 mg, 0.10 mmol) in a 5 mL stainless steel jar along with 20.0 μL of ethanol, and two stainless steel balls 5 mm in diameter and 0.50 g mass for 30 minutes in a Retsch MM200 shaker mill operating at 25 Hz.

2. Additional mechanochemical experiments

Table S1. Summary of parameters and qualitative outcomes of additional mechanochemical experiments

stoichiometric ratio	*m* (*cis*-$CoCl_2$**bzpy**$_2$) / mg	*m* (**14tfib**) / mg	volume of ethanol / µL	time / min	jar type	balls	milling frequency / Hz	outcome
1:1	55.3	44.8	20	30	5 mL stainless steel	two stainless steel (*d*=5 mm, *m*= 0.50 g)	25	(*cis*-$CoCl_2$**bzpy**$_2$)(**14tfib**)$_2$ + (*cis*-$CoCl_2$**bzpy**$_2$)
1:1	55.3	44.8	20	60	5 mL stainless steel	two stainless steel (*d*=5 mm, *m*= 0.50 g)	25	(*trans*-$CoCl_2$**bzpy**$_2$)(**14tfib**)$_2$ + (*cis*-$CoCl_2$**bzpy**$_2$)
1:2	38.2	61.8	20	30	5 mL stainless steel	two stainless steel (*d*=5 mm, *m*= 0.50 g)	25	(*cis*-$CoCl_2$**bzpy**$_2$)(**14tfib**)$_2$ *
1:2	38.2	61.8	20	10	14 mL teflon	teflon-coated ball with steel core (*d*=12 mm, *m*=3.32 g)	20	(*trans*-$CoCl_2$**bzpy**$_2$)(**14tfib**)$_2$
1:2	38.2	61.8	20	5	5 mL stainless steel	two stainless steel (*d*=5 mm, *m*= 0.50 g)	10	(*trans*-$CoCl_2$**bzpy**$_2$)(**14tfib**)$_2$ + (*cis*-$CoCl_2$**bzpy**$_2$) + **14tfib**
1:2	38.2	61.8	20	20	5 mL stainless steel	two stainless steel (*d*=5 mm, *m*= 0.50 g)	25	(*trans*-$CoCl_2$**bzpy**$_2$)(**14tfib**)$_2$
1:2	38.2 + seed 0.6 mg (*cis*-$CoCl_2$**bzpy**$_2$)(**14tfib**)$_2$	61.8	20	30	5 mL stainless steel	two stainless steel (*d*=5 mm, *m*= 0.50 g)	25	(*trans*-$CoCl_2$**bzpy**$_2$)(**14tfib**)$_2$
1:2	38.2 + seed 0.6 mg (*cis*-$CoCl_2$**bzpy**$_2$)(**14tfib**)$_2$	61.8	20	20	5 mL stainless steel	two stainless steel (*d*=5 mm, *m*= 0.50 g)	25	(*trans*-$CoCl_2$**bzpy**$_2$)(**14tfib**)$_2$
-	55.4	-	40	60	5 mL stainless steel	one stainless steel (*d*=7 mm, *m*=1.29 g)	25	*cis*-$CoCl_2$**bzpy**$_2$
-	70.0	-	-	30	5 mL stainless steel	two stainless steel (*d*=5 mm, *m*= 0.50 g)	25	*cis*-$CoCl_2$**bzpy**$_2$

* This experiment was not reproducible. Repeated experiments yielded (*trans*-$CoCl_2$**bzpy**$_2$)(**14tfib**)$_2$.

An additional one-pot mechanochemical experiment was performed by kneading of $CoCl_2\cdot 6H_2O$ (16.9 mg, 0.10 mmol), **bzpy** (26.0 mg, 0.20 mmol) and halogen bond donor **14tfib** (57.1 mg, 0.20 mmol) in a mortar for 10 min alongside with gradually adding 40 µL of ethanol. Product of the reaction was (*cis*-$CoCl_2$**bzpy**$_2$)(**14tfib**) and excess of **14tfib.**

3. Powder X-ray diffraction experiments

The experiments for analysis of products of mechanochemical synthesis were performed on a PHILIPS PW 1840 X-ray diffractometer with Cu$K_{\alpha1,2}$ radiation (1.54056 Å) at 40 mA and 40 kV. The scattered intensities were measured with a scintillation counter. The angular range was from 3 or 5 to 40° (2θ) with a continuous step size of 0.03°, and a measuring time of 0.3 s per step. Data collection and analysis were performed using the program package X'Pert HighScore Plus.[45]

graf 1

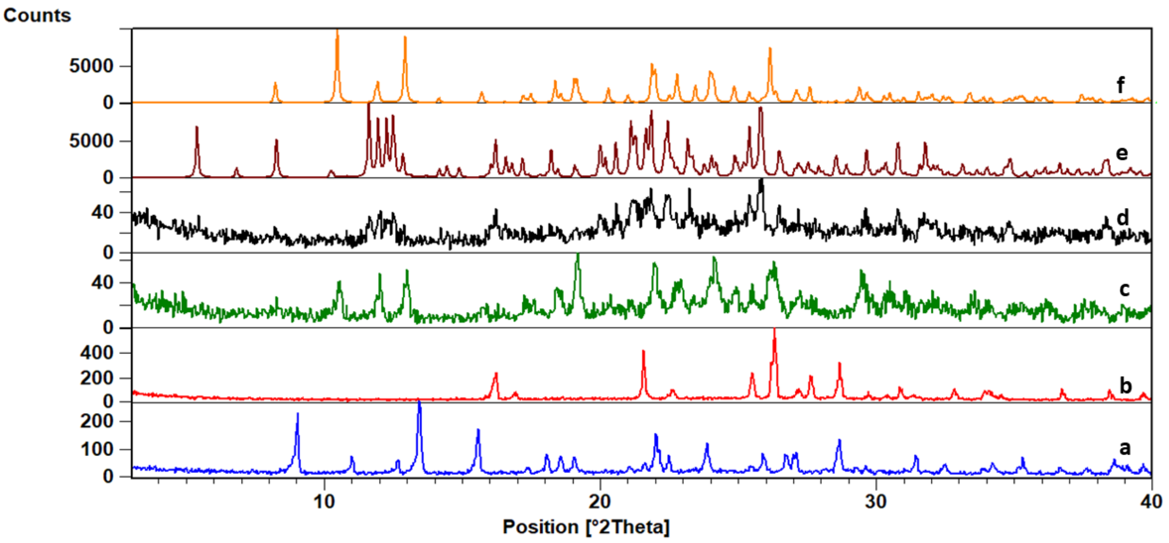

Figure S1. Measured PXRD patterns (λ = 1.54056 Å) for: a) *cis*-$CoCl_2$**bzpy**$_2$; b) **14tfib**; c) product of LAG of *cis*-$CoCl_2$**bzpy**$_2$ and **14tfib** in a 1:2 molar ratio using a 5 mL stainless steel jar along with 20.0 µL EtOH, and two stainless steel balls 5 mm in diameter (0.50 g mass) for 30 minutes in a Retsch MM200 shaker mill operating at 25 Hz, d) product of kneading *cis*-$CoCl_2$**bzpy**$_2$ and **14tfib** in a 1:2 molar ratio, using a mortar for 15 minutes, adding 120 µL of EtOH in small portions, e) calculated PXRD pattern of (*cis*-$CoCl_2$**bzpy**$_2$)(**14tfib**)$_2$, f) calculated PXRD pattern of (*trans*-$CoCl_2$**bzpy**$_2$)(**14tfib**)$_2$.

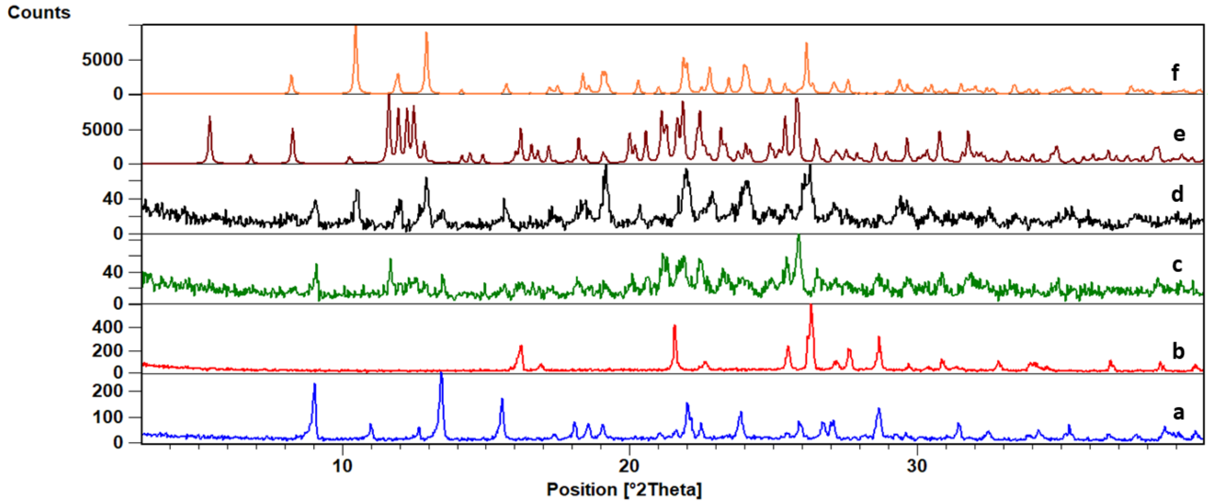

Figure S2. Measured PXRD patterns (λ = 1.54056 Å) of: a) *cis*-CoCl$_2$**bzpy**$_2$, b) **14tfib**, c) product obtained by grinding a mixture with a 1:1 molar ratio *cis*-CoCl$_2$**bzpy**$_2$ to **14tfib** in a 5 mL stainless steel jar along with 20.0 μL of ethanol, and two stainless steel balls 5 mm in diameter and 0.50 g mass for 30 minutes in a Retsch MM200 Shaker Mill operating at 25 Hz, d) product obtained by grinding a mixture with a 1:1 molar ratio *cis*-CoCl$_2$**bzpy**$_2$ to **14tfib** in a 5 mL stainless steel jar along with 20.0 μL of ethanol, and two stainless steel balls 5 mm in diameter and 0.50 g mass for 60 minutes in a Retsch MM200 shaker mill operating at 25 Hz, e) calculated PXRD pattern of (*cis*-CoCl$_2$**bzpy**$_2$)(**14tfib**)$_2$, f) calculated PXRD pattern of (*trans*-CoCl$_2$**bzpy**$_2$)(**14tfib**)$_2$.

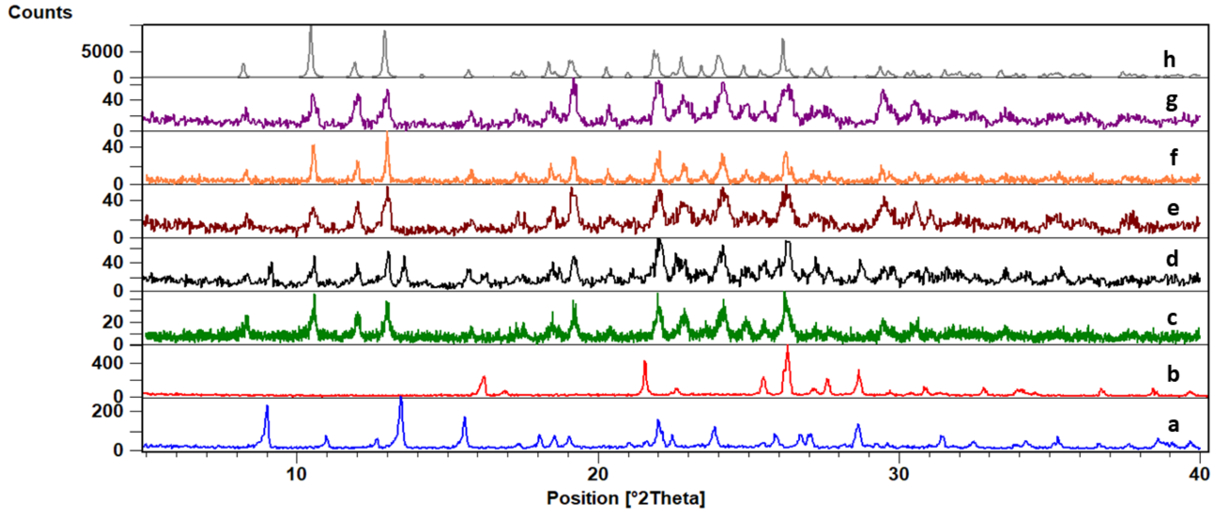

Figure S3. Measured PXRD patterns (λ = 1.54056 Å) of: a) *cis*-CoCl$_2$**bzpy**$_2$, b) **14tfib**, products obtained by grinding a mixture with a 1:2 molar ratio *cis*-CoCl$_2$**bzpy**$_2$ to **14tfib**: c) in a 14 mL Teflon jar along with 20.0 μL EtOH, and a Teflon ball 12 mm in diameter and 3.32 g mass for 10 minutes in a Retsch MM200 shaker mill operating at 20 Hz, d) in a 5 mL stainless steel jar along with 20.0 μL EtOH, and two stainless steel balls 5 mm in diameter and 0.50 g mass for 5 minutes in a Retsch MM200 shaker mill operating at 10 Hz, e) in a 5 mL stainless steel jar along with 20.0 μL EtOH, and two stainless steel balls 5 mm in diameter and 0.50 g mass for 20 minutes in a Retsch MM200 shaker mill operating at 25 Hz, f) in a 5 mL stainless steel jar along with 20.0 μL EtOH, seeds of (*cis*-CoCl$_2$**bzpy**$_2$)(**14tfib**)$_2$, two stainless steel balls 5 mm in diameter and 0.50 g mass for 30 minutes in a Retsch MM200 shaker mill operating at 25 Hz, g) in a 5 mL stainless steel jar along with 20.0 μL EtOH, seeds of (*cis*-CoCl$_2$**bzpy**$_2$)(**14tfib**)$_2$, two stainless steel balls 5 mm in diameter and 0.50 g mass for 20 minutes in a Retsch MM200 shaker mill operating at 25 Hz frequency, h) calculated PXRD pattern of (*trans*-CoCl$_2$**bzpy**$_2$)(**14tfib**)$_2$.

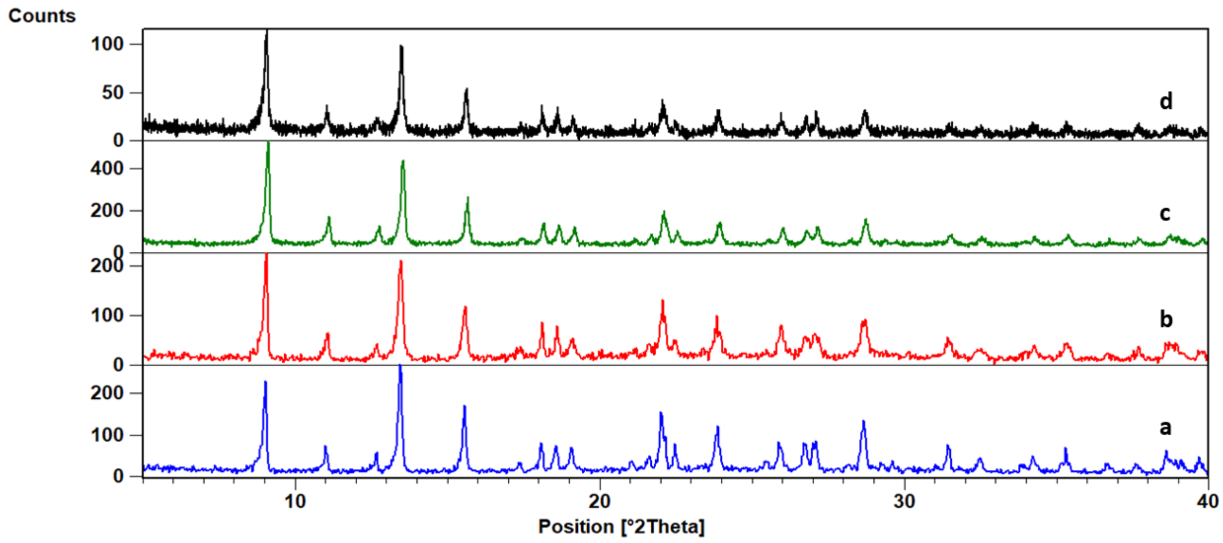

Figure S4. Measured PXRD patterns (λ = 1.54056 Å) of: a) *cis*-$CoCl_2$**bzpy**$_2$, b) products obtained by grinding 70.0 mg *cis*-$CoCl_2$**bzpy**$_2$ in a 5 mL stainless steel jar along with two stainless steel balls 5 mm in diameter and 0.50 g mass for 30 minutes in a Retsch MM200 Shaker Mill operating at 25 Hz, c) products obtained by grinding 55.4 mg *cis*-$CoCl_2$**bzpy**$_2$ in a 5 mL stainless steel jar along with 40.0 µL EtOH, one stainless steel ball 7 mm in diameter and 1.39 g mass for 60 minutes in a Retsch MM200 shaker mill operating at 25 Hz, d) products obtained by grinding 90 mg *cis*-$CoCl_2$**bzpy**$_2$ in a 5 mL stainless steel jar along with 40.0 µL 2,2,2-trifluorethanol, two stainless steel balls 5 mm in diameter and 0.50 g mass for 30 minutes in a Retsch MM200 shaker mill operating at 25 Hz.

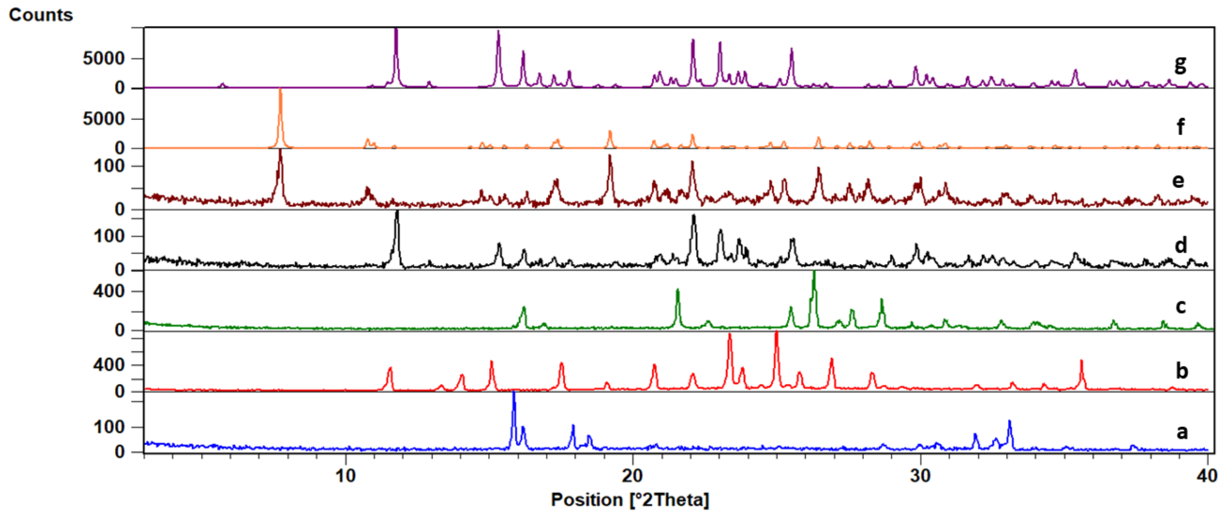

Figure S5. Measured PXRD patterns (λ = 1.54056 Å) of: a) $CoCl_2 \cdot 6H_2O$, b) **bzpy,** c) **14tfib**, d) products of grinding a mixture with a 1:2:1 molar ratio of $CoCl_2 \cdot 6H_2O$ to **bzpy** to **14tfib** in a 5 mL stainless steel jar along with 20.0 µL EtOH, and two stainless steel balls 5 mm in diameter and 0.50 g mass for 30 minutes in a Retsch MM200 shaker mill operating at 25 Hz, e) products obtained by kneading a mixture with a 1:2:1 molar ratio $CoCl_2 \cdot 6H_2O$ to **bzpy** to **14tfib** in a mortar for 10 minutes adding 40.0 µL EtOH in small portions, f) calculated PXRD pattern of (*cis*-$CoCl_2$**bzpy**$_2$)(**14tfib**), g) calculated PXRD pattern of (*trans*-$CoCl_2$**bzpy**$_2$)(**14tfib**).

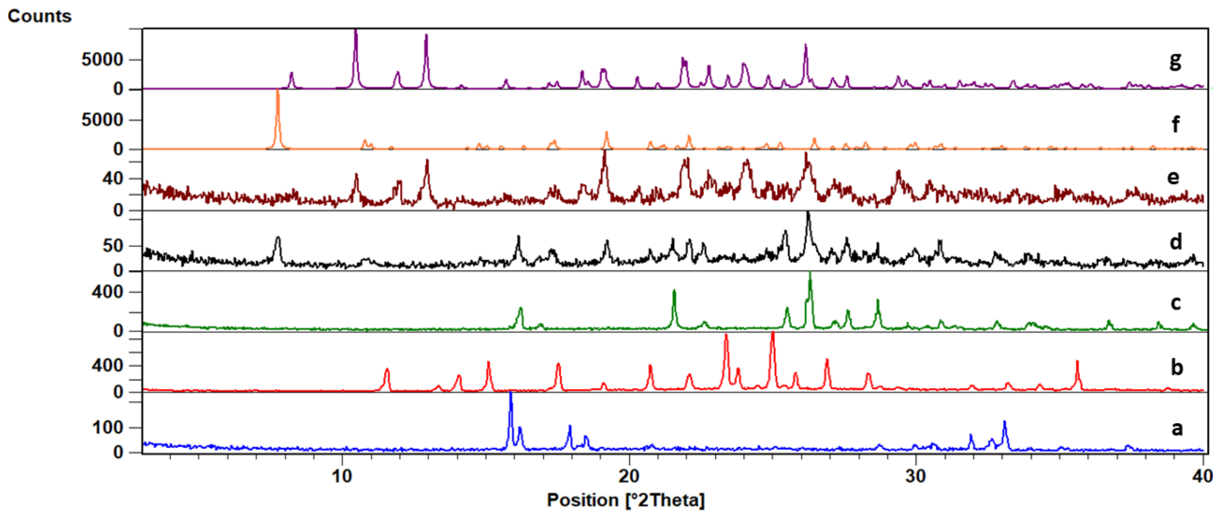

Figure S6. Measured PXRD patterns (λ = 1.54056 Å) of: a) $CoCl_2{\cdot}6H_2O$, b) **bzpy,** c) **14tfib**, d) products obtained by grinding a mixture with a 1:2:2 molar ratio of $CoCl_2{\cdot}6H_2O$ to **bzpy** to **14tfib** in mortar for 10 minutes adding 40.0 µL of EtOH in small portions,, e) products of grinding a mixture with a 1:2:2 molar ratio of $CoCl_2{\cdot}6H_2O$ to **bzpy** to **14tfib** in a 5 mL stainless steel jar along with 20.0 µL EtOH, and two stainless steel balls 5 mm in diameter and 0.50 g mass for 60 minutes in a Retsch MM200 shaker mill operating at 25 Hz frequency, f) calculated PXRD pattern of (*cis*-$CoCl_2$**bzpy**$_2$)(**14tfib**), g) calculated PXRD pattern of (*trans*-$CoCl_2$**bzpy**$_2$)(**14tfib**)$_2$.

4. Crystallization

Single crystals for SCXRD of (*cis*-$CoCl_2$**bzpy**$_2$)(**14tfib**)$_2$ were isolated from a solution obtained by dissolving a mixture of $CoCl_2{\cdot}6H_2O$ (20.0 mg, 0.084 mmol), **bzpy** (30.8 mg, 0.17 mmol) and **14tfib** (33.8 mg, 0.084 mmol) in 1.25 mL of a hot mixture of ethanol and 2,2,2-trifluorethanol (volume ratio 3:2) and leaving the solution to cool and evaporate at room temperature for 3 days.

Single crystals for SCXRD of (*cis*-$CoCl_2$**bzpy**$_2$)(**14tfib**) were isolated from a solution obtained by dissolving a mixture of $CoCl_2{\cdot}6H_2O$ (10.0 mg, 0.042 mmol), **bzpy** (15.4 mg, 0.085 mmol) and **14tfib** (33.8 mg, 0.084 mmol) in 0.5 mL of ethanol and leaving the solution to evaporate at room temperature for 1 hour.

Single crystals for SCXRD of (*trans*-$CoCl_2$**bzpy**$_2$)(**14tfib**)$_2$ were isolated from a solution obtained by dissolving a mixture of $CoCl_2{\cdot}6H_2O$ (40.0 mg, 0.17 mmol), **bzpy** (61.6 mg, 0.34 mmol) and **14tfib** (67.6 mg, 0.17 mmol) in 2.5 mL of a hot mixture of ethanol and 2,2,2-trifluorethanol (volume ratio 3:2) and leaving the solution to cool and evaporate at room temperature for 3 days.

Single crystals for SCXRD of *cis*-$CoCl_2$**bzpy**$_2$ were obtained by dissolving a mixture of $CoCl_2\cdot 6H_2O$ (40.0 mg, 0.081 mmol) and **bzpy** (61.6 mg, 0.162 mmol) in 0.7 mL of ethanol and leaving the solution to evaporate at room temperature for 1 day.

Additional crystallization experiments for determining possibility of *cis-trans* isomerisation in the solution were made by dissolving either 25.0 mg of *cis*-$CoCl_2$**bzpy**$_2$, or 40.0 mg $CoCl_2(H_2O)_6$ + 61.6 mg **bzpy** in the chosen solvents. Yielded crystals were analysed by PXRD. Experimental conditions and outcomes are summed up in the following table.

Table S2 Experimental conditions and outcomes of crystallization experiments.

experiment nb.	starting material	solvent	*V* / mL	heating[a] (yes/no)	outcome
1	*cis*-$CoCl_2$**bzpy**$_2$	ethanol	4 mL	no	*cis*-$CoCl_2$**bzpy**$_2$
2	*cis*-$CoCl_2$**bzpy**$_2$	ethanol	4 mL	yes	*cis*-$CoCl_2$**bzpy**$_2$
3	*cis*-$CoCl_2$**bzpy**$_2$	2,2,2-trifluorethanol	3 mL	no	*cis*-$CoCl_2$**bzpy**$_2$
4	*cis*-$CoCl_2$**bzpy**$_2$	2,2,2-trifluorethanol	3 mL	no	*cis*-$CoCl_2$**bzpy**$_2$
5	*cis*-$CoCl_2$**bzpy**$_2$	2,2,2-trifluorethanol	2 mL	yes	*cis*-$CoCl_2$**bzpy**$_2$
6	*cis*-$CoCl_2$**bzpy**$_2$	acetonitrile	4 mL	no	*cis*-$CoCl_2$**bzpy**$_2$
7	*cis*-$CoCl_2$**bzpy**$_2$	methanol	4 mL	no	*cis*-$CoCl_2$**bzpy**$_2$
8	$CoCl_2(H_2O)_6$ + **bzpy**	ethanol	0.8 mL	yes	*cis*-$CoCl_2$**bzpy**$_2$
9	$CoCl_2(H_2O)_6$ + **bzpy**	tetrahydrofuran	2.6 mL	yes	*cis*-$CoCl_2$**bzpy**$_2$
10	$CoCl_2(H_2O)_6$ + **bzpy**	tetrahydrofuran	2.6 mL	yes + ultrasound	*cis*-$CoCl_2$**bzpy**$_2$
11	$CoCl_2(H_2O)_6$ + **bzpy**	tetrahydrofuran	2.0 mL	yes + ultrasound	*cis*-$CoCl_2$**bzpy**$_2$
12	$CoCl_2(H_2O)_6$ + **bzpy**	acetonitrile	1.6 mL	yes + ultrasound	*cis*-$CoCl_2$**bzpy**$_2$

[a]crystallisation mixtures were heated close to boiling.

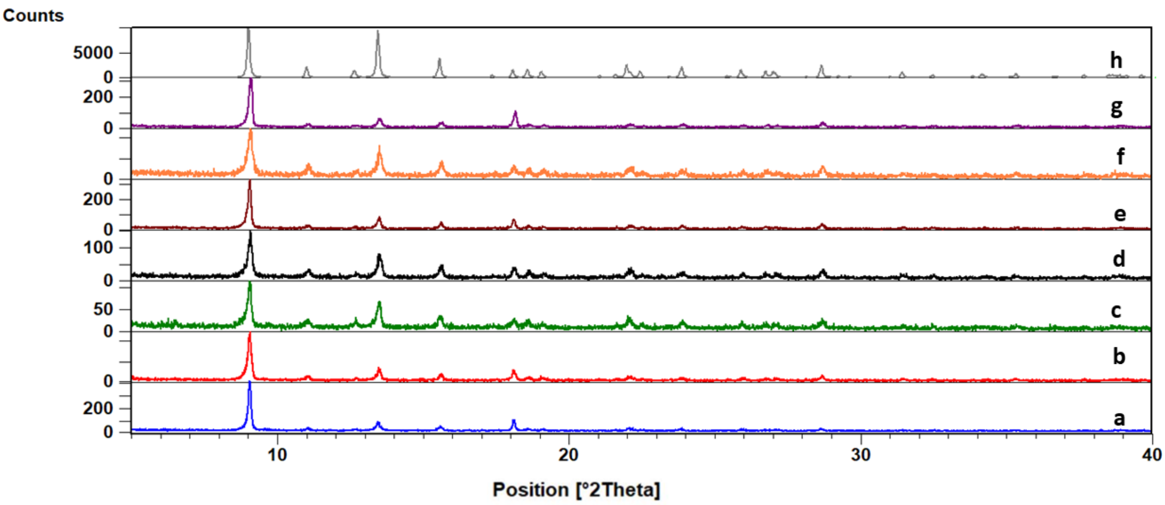

Figure S7. Measured PXRD patterns (λ = 1.54056 Å) of crystals yielded from recrystallization experiments of *cis*-$CoCl_2$**bzpy**$_2$: a) exp. nb. 1, b) exp. nb. 2, c) exp. nb. 3, d) exp. nb. 4, e) exp. nb. 5, f) exp. nb. 6, g) exp. nb. 7, h) calculated PXRD pattern of *cis*-$CoCl_2$**bzpy**$_2$.

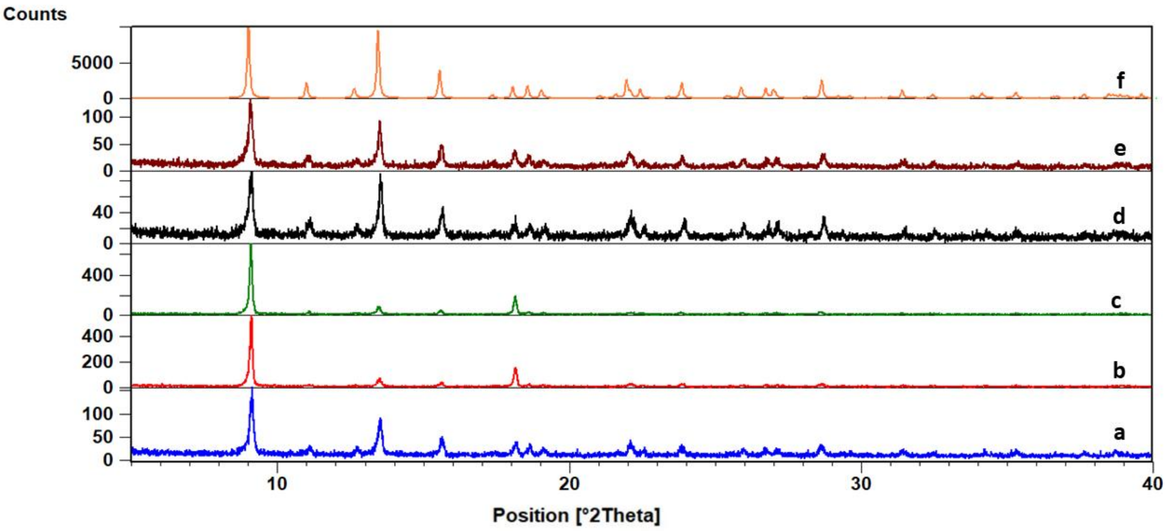

Figure S8. Measured PXRD patterns (λ = 1.54056 Å) of crystals yielded from crystallization experiments of $CoCl_2(H_2O)_6$ and **bzpy**: a) exp. nb. 8, b) exp. nb. 9, c) exp. nb. 10, d) exp. nb. 11, e) exp. nb. 12, f) calculated PXRD pattern of *cis*-$CoCl_2$**bzpy**$_2$.

5. Crystal structure determination

Single crystal X-ray diffraction experiments were performed at 295 K on an Oxford Diffraction Xcalibur diffractometer equipped with a Sapphire3 CCD detector, using graphite-monochromated Mo$K\alpha$ (λ = 0.71073 Å) radiation. Programs CrysAlis CCD[46] and CrysAlis RED[47] were used for data collection, cell refinement and data reduction. All structures were solved and refined using the SHELXT[48] and SHELXL[49] programs, respectively. Structural refinement was performed on F^2 using all data. All aryl hydrogen atoms were placed at calculated positions and treated as riding on their parent atoms. All calculations were

performed using the WinGX crystallographic suite of programs.[50] The molecular structures of compounds were prepared by Mercury.[51]

Powder X-ray diffraction measurement for indexing and structure solution of cocrystal (*trans*-$CoCl_2$**bzpy**$_2$)(**14tfib**) was performed on a Stoe Transmission Powder Diffractometer (STADI-P, STOE & CIE) with Mo$K\alpha_1$ radiation, equipped with an array of three MYTHEN 1K detectors (Dectris Ltd.), and a Ge(111) Johann-type monochromator (STOE & CIE). During data collection only one Mythen 1K detector was used. The sample was gently ground, filled into a 0.5 mm diameter glass capillary, and spun during data collection for better particle statistics.

Indexing of the X-ray diffractogram for (*trans*-$CoCl_2$**bzpy**$_2$)(**14tfib**) was carried out by an iterative use of singular value decomposition using the program TOPAS V5,[52] leading to a triclinic space group with the lattice parameters listed in Table 1. The peak profile was determined by a Pawley refinement using the fundamental parameter approach as implemented in TOPAS. The background was modeled by a Chebychev polynomial of 11th order. The crystal structure was determined using the global optimization method of simulated annealing (SA). Three rigid bodies in z-matrix notation with idealized bond lengths and angles from related single crystal structures were used during the *ab initio* structure solution process and for the consequent Rietveld refinement. The second linker, phenyl-2-pyridylketone (**bzpy**) was decomposed into two rigid bodies with phenyl and a 2-pyridylketone subunit using a distance restrain between the two rigid bodies of 1.5 Å (C9-C10). 1,4-diiodotetrafluorobenzene (**14tfib**) was described by a rigid body in z-matrix notation including a dummy atom in the center of the ring, which was placed on a center of inversion (0, 0, 0). For the final Rietveld refinement, the background, lattice parameters, microstructure in form of crystallite size (Gaussian) and microstrain (Gaussian and Lorentzian component), most translations and rotations of the rigid bodies were refined without any constraint for the final refinement. Only the translational components of **14tfib** were constrained, so that a center of inversion is in the center of the molecule.

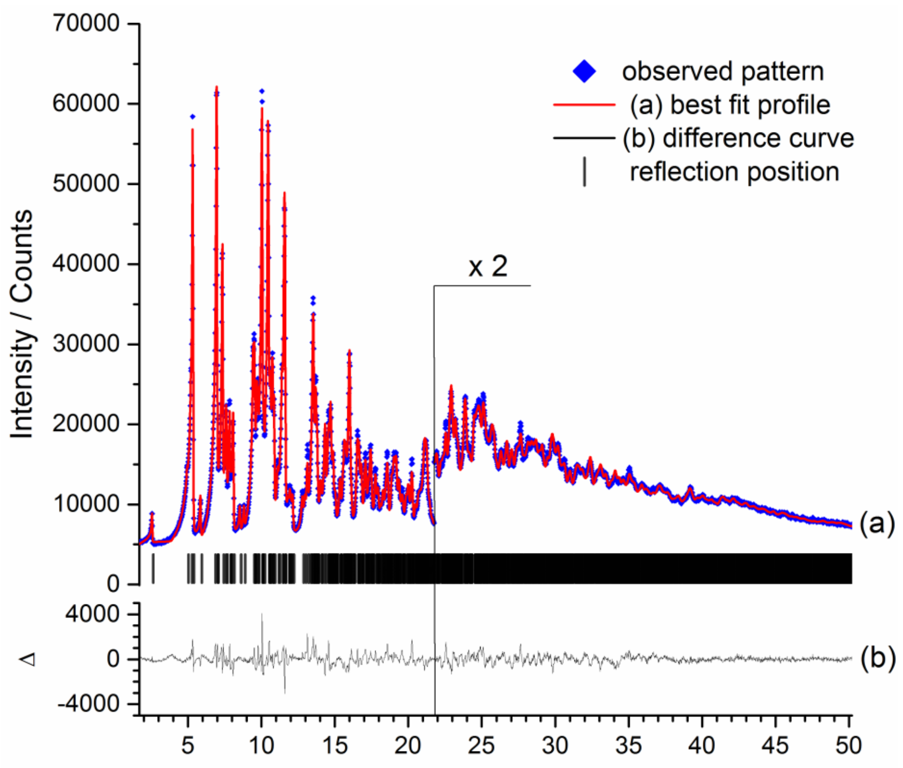

Figure S9. Difference plot of the Rietveld refinement of (*trans*-$CoCl_2$**bzpy**$_2$)(**14tfib**) (λ = 1.54056 Å) with the observed pattern (blue diamonds), calculated pattern (red line), calculated peak positions (dashes), and the difference curve (black line). The higher scattering region (2θ > 22 °) was enlarged by a factor of 2 for better visualization.

Table S3. General and crystallographic data for the prepared compounds.

	cis-$CoCl_2$**bzpy**$_2$	(*cis*-$CoCl_2$**bzpy**$_2$)(**14tfib**)$_2$	(*cis*-$CoCl_2$**bzpy**$_2$)(**14tfib**)
Molecular formula	($CoCl_2C_{24}H_{18}N_2O_2$)	($CoCl_2C_{24}H_{18}N_2O_2$) ($C_6F_4I_2$)$_2$	($CoCl_2C_{24}H_{18}N_2O_2$) ($C_6F_4I_2$)
M_r	496.23	1299.95	898.09
Crystal system	orthorhombic	monoclinic	monoclinic
Space group	*Pbcn*	$P2_1/n$	$P2/c$
Crystal data:			
a / Å	14.0203(7)	8.8731(4)	12.0749(7)
b / Å	8.0548(5)	32.8016(16)	11.3956(7)
c / Å	19.6213(9)	14.1022(8)	12.5427(8)
α / °	90	90	90
β / °	90	91.807(5)	110.169(7)
γ / °	90	90	90
V / Å^3	2215.9(2)	4102.4(4)	1620.05(18)
Z	4	4	2
D_{calc} / g cm^{-3}	1.487	2.105	1.841
λ(MoK_α) / Å	0.71073	0.71073	0.71073
T / K	295	295	295
Crystal size / mm^3	0.46 × 0.52 × 0.22	0.70 × 0.30 × 0.15	0.75 × 0.63 × 0.45
μ / mm^{-1}	1.039	3.630	2.653
F(000)	1012	2436	862
Refl. collected/unique	2166/1430	7171/4722	3707/2596
Data/restraints/parameters	141	496	195
$\Delta\rho_{max}$, $\Delta\rho_{min}$ / e Å^{-3}	0.452; –0.285	0.643; −0.736	0.752; −0.575
$R[F^2>4\sigma(F^2)]$	0.0264	0.0408	0.0279
$wR(F^2)$	0.0660	0.0863	0.0613
Goodness-of-fit, S	0.866	1.049	0.889
Technique	SCXRD	SCXRD	SCXRD

	(*trans*-$CoCl_2$**bzpy**$_2$)(**14tfib**)$_2$		(*trans*-$CoCl_2$**bzpy**$_2$)(**14tfib**)
Molecular formula	($CoCl_2C_{24}H_{18}N_2O_2$)($C_6F_4I_2$)$_2$	Molecular formula	($CoCl_2C_{24}H_{18}N_2O_2$)($C_6F_4I_2$)
M_r	1299.95	M_r	898.09
Crystal system	triclinic	Crystal system	triclinic
Space group	$P\bar{1}$	Space group	$P\bar{1}$
Crystal data:			
a / Å	9.257(5)	a / Å	6.0886(2)
b / Å	10.357(5)	b / Å	8.4410(3)
c / Å	11.670(5)	c / Å	15.7482(6)
α / °	88.054(5)	α / °	93.424(3)
β / °	66.993(5)	β / °	100.108(3)
γ / °	84.081(5)	γ / °	105.483(2)
V / Å^3	1024.3(9)	V / Å^3	763.022
Z	1	Z	1
D_{calc} / g cm^{-3}	2.107	D_{calc} / g cm^{-3}	1.955
λ(MoK_α) / Å	0.71073	λ(MoK_α) / Å	0.7093
T / K	295	T / K	295
Crystal size / mm^3	0.68 × 0.47 × 0.28	μ / mm^{-1}	2.828
μ / mm^{-1}	3.634	R_{wp} /%[a]	2.72
F(000)	609	R_p/%[a]	2.09
Refl. collected/unique	3567/ 2648	R_{exp}/%[a]	1.02
Data/restraints/parameters	250	R_{Bragg}[a]	1.15
$\Delta\rho_{max}$, $\Delta\rho_{min}$ / e Å^{-3}	0.727; –0.844	Starting angle 2θ / °	1.7
$R[F^2>4\sigma(F^2)]$	0.0292	Final angle 2θ / °	50.190
$wR(F^2)$	0.0665	Step width 2θ / °	0.0075
Goodness-of-fit, S	0.949	Time per scan / h	12
		No. of variables	45
Technique	SCXRD		PXRD

[a] R_{wp}, R_p, R_{exp}, R_{Bragg} as defined in TOPAS.

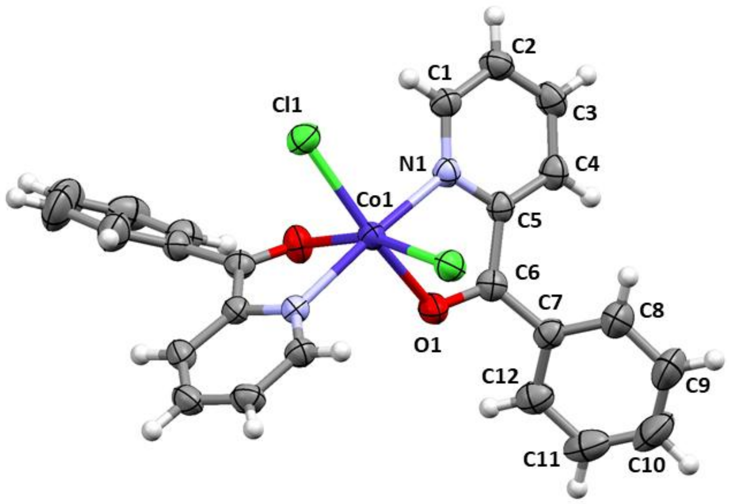

Figure S10. Molecular structure of *cis*-$CoCl_2$**bzpy**$_2$ showing the atom-labelling scheme. Displacement ellipsoids of non-hydroge atoms are drawn at the 50 % probability level, while hydrogen atoms are shown as spheres of arbitrary radius.

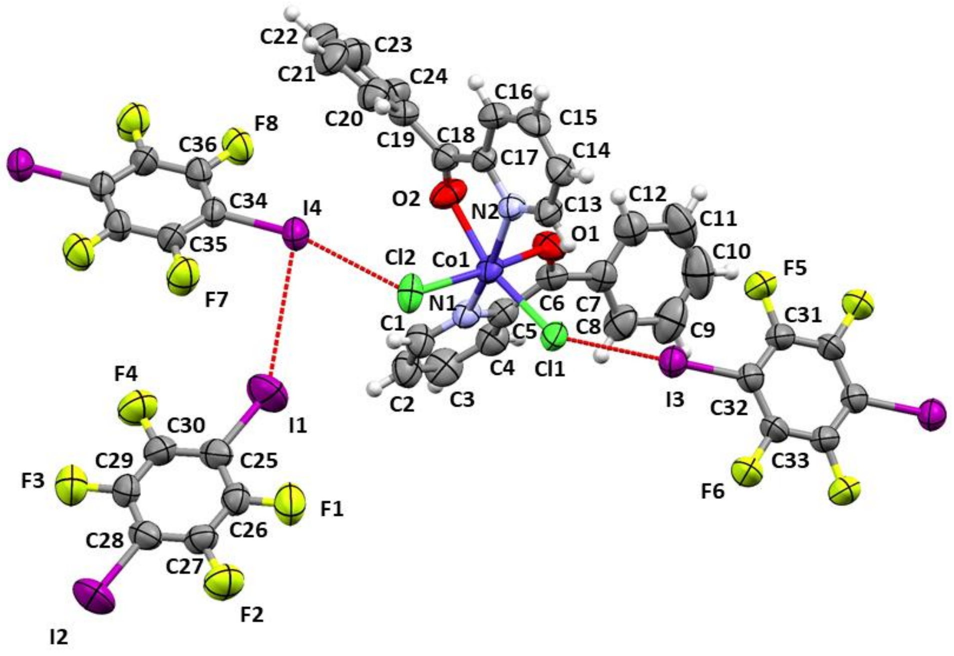

Figure S11. Molecular structure of (*cis*-$CoCl_2$**bzpy**$_2$)(**14tfib**)$_2$ showing the atom-labelling scheme. Displacement ellipsoids of non-hydrogen atoms are drawn at the 50 % probability level, halogen bonds are marked with red dashed lines, and hydrogen atoms are shown as spheres of arbitrary radius.

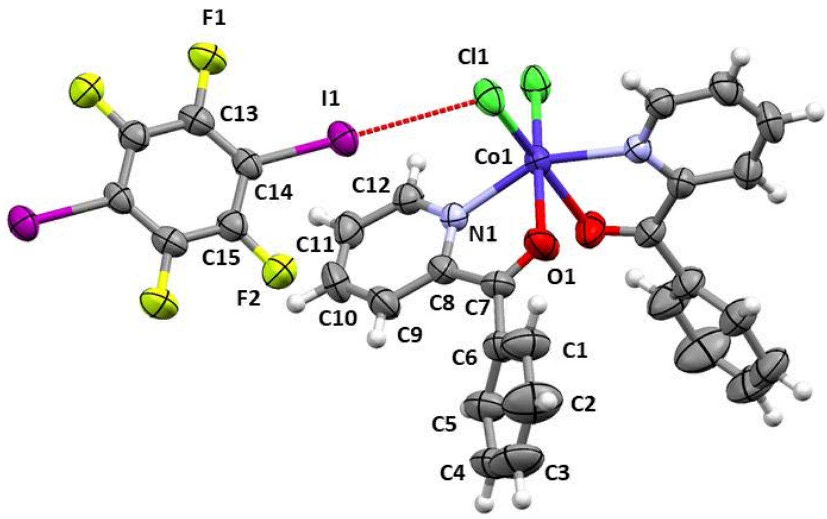

Figure S12. Molecular structure of (*cis*-$CoCl_2$**bzpy**$_2$)(**14tfib**) showing the atom-labelling scheme. Displacement ellipsoids of non-hydrogen atoms are drawn at the 50 % probability level, halogen bond is marked with red dashed lines, and hydrogen atoms are shown as spheres of arbitrary radius.

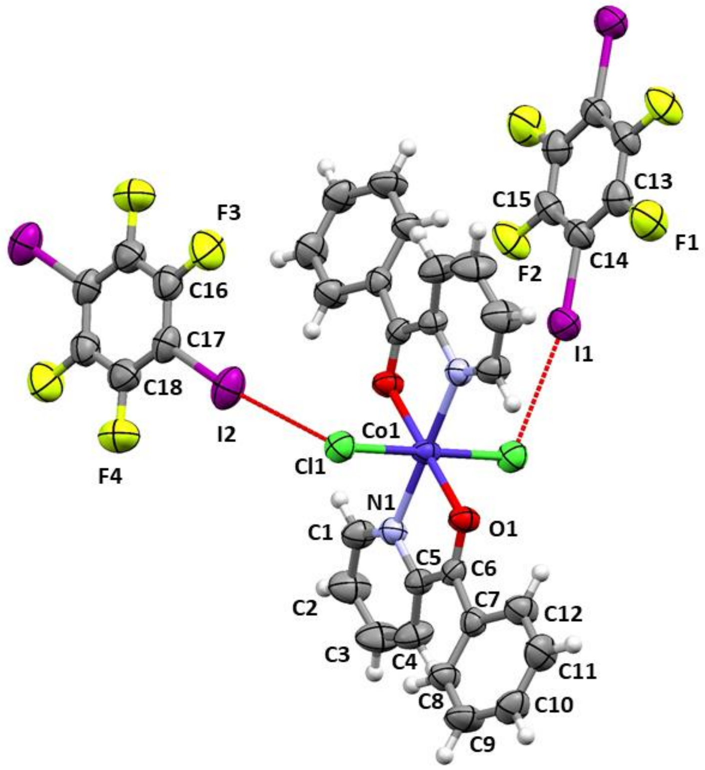

Figure S13. Molecular structure of (*trans*-$CoCl_2$**bzpy**$_2$)(**14tfib**)$_2$ showing the atom-labelling scheme. Displacement ellipsoids of non-hydrogen atoms are drawn at the 50% probability level, halogen bonds are marked with red dashed lines, and hydrogen atoms are shown as spheres of arbitrary radius.

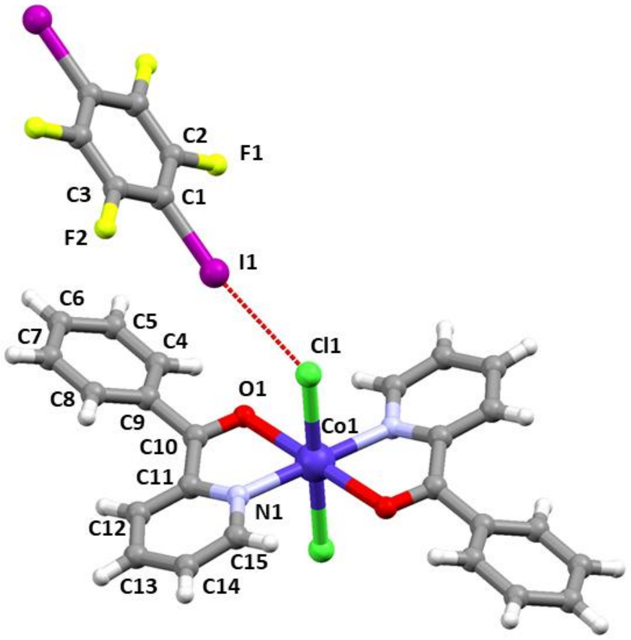

Figure S14. Molecular structure of (*trans*-$CoCl_2$**bzpy**$_2$)(**14tfib**) showing the atom-labelling scheme.

6. *In situ* PXRD monitoring

In situ monitoring of mechanochemical reactions were performed at the Powder Diffraction and Total Scattering beamline P02.1 at DESY, Hamburg. The multicomponent reaction mixtures (300 mg) were placed along with two stainless steel balls, 7 mm in diameter and 1.39 g weight in X-ray transparent 15 mL poly(methyl methacrylate) jars, EtOH was added (in such a way that the liquid and solids mixed only after the experiment started), and the mixture milled on a modified Retsch MM400 vibration mill at a frequency of 25 Hz. Diffraction patterns were collected with an exposure time of 10 s, using a 60 keV X-ray beam (λ = 0.20709 Å) and a Perkin Elmer 2D area detector. All 2D PXRD patterns were subsequently integrated into 1D PXRD patterns using Fit2D or Dioptas.

Table S4. Parametars of *in situ* monitored reactions.

**nb.**	milling mixture	liquid	V / µL	t / min
1	165,8 mg (0.33 mmol) *cis*-$CoCl_2$**bzpy**$_2$ + 134,2 mg (0.33 mmol) **14tfib**	ethanol	10	40
2	114,5 mg (0.23 mmol) *cis*-$CoCl_2$**bzpy**$_2$ + 185,5 mg (0.46 mmol) **14tfib**	ethanol	20	20

Sequential Rietveld refinements were performed using TOPAS V5.[52] A silicon standard measurement was used to describe the instrumental profile function (IPF) by applying equivalent conditions (same milling frequency and exposure time). The IPF was described using $Z/\cos^2(\theta)$ of the Gaussian profile and $X\cdot\tan(\theta)$ of the Lorentzian profile from the modified Thompson-Cox-Hasting pseudo-Voight function as implemented in TOPAS. The

crystal structure of **14tfib** (CSD refcode ZZZAVM02) was obtained from the CSD database, the other crystal structures were solved from single crystal X-ray diffraction (SCXRD) or PXRD data. Their microstructure and lattice parameter were refined individually with one *in situ* pattern, where the phase was observed with high abundance and fixed for subsequent sequential Rietveld refinement. During the sequential Rietveld refinement, only the scale parameters of all phases and background were freely refined.

7. Thermal analysis

The TGA-DSC measurements were performed on a Mettler-Toledo TGA-DSC 3+ module. Samples were placed in open 70 μL aluminium oxide crucibles heated from 25 to 600 °C at a rate of 10 °C min^{-1} under nitrogen flow of 50 mL min^{-1}. Data collection and analysis were performed using the program package STARe Software v16.20.[53]

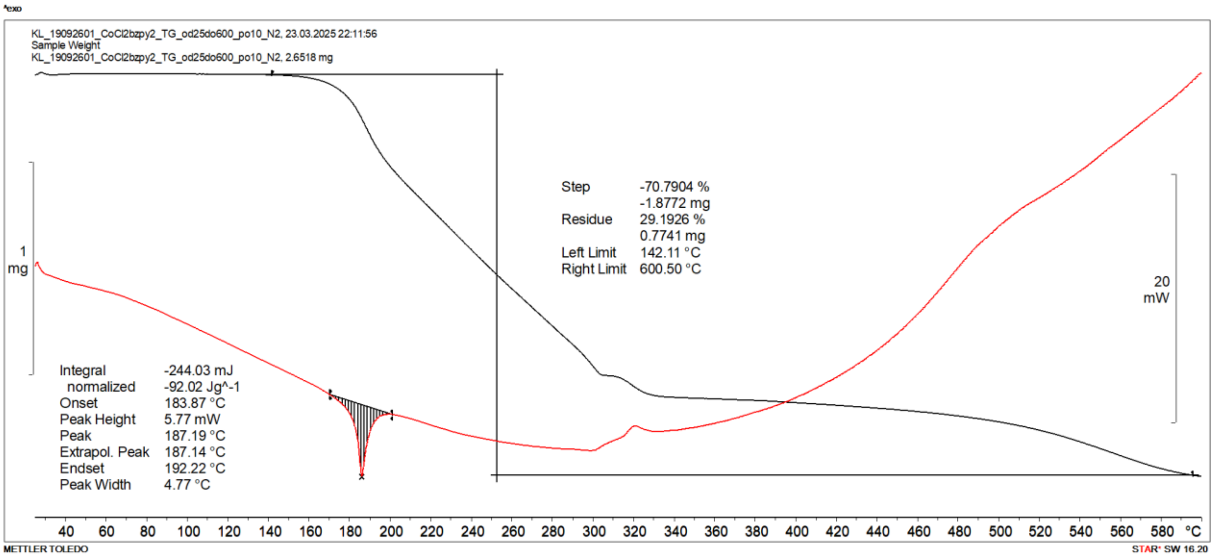

Figure S15. TGA (black curve) and DSC (red curve) thermograms for *cis*-$CoCl_2$**bzpy**$_2$.

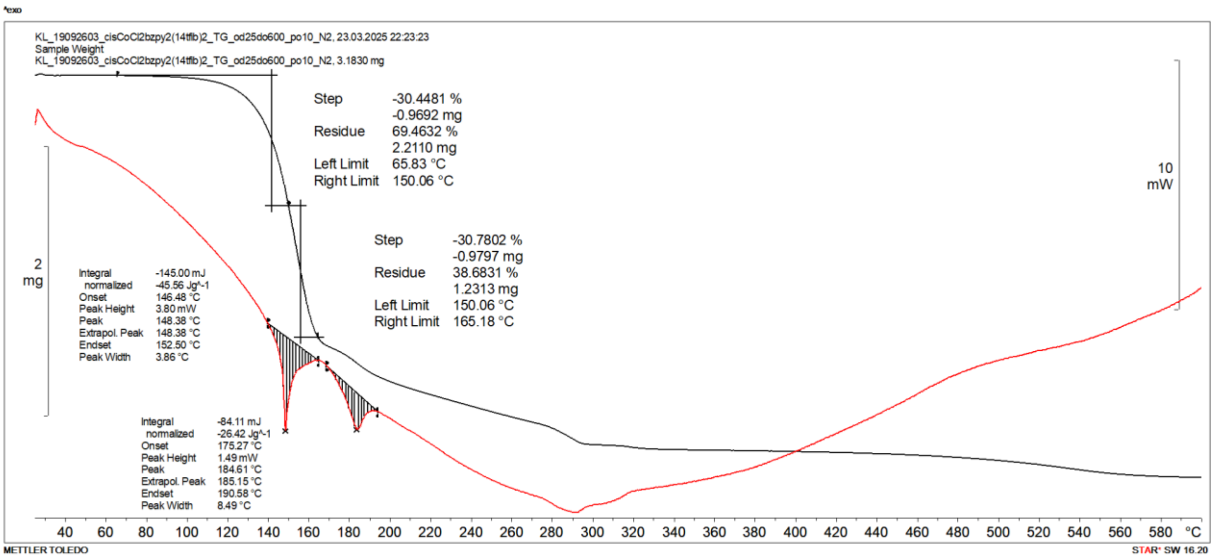

Figure S16. TGA (black curve) and DSC (red curve) thermograms for (*cis*-$CoCl_2$**bzpy**$_2$)(**14tfib**)$_2$.

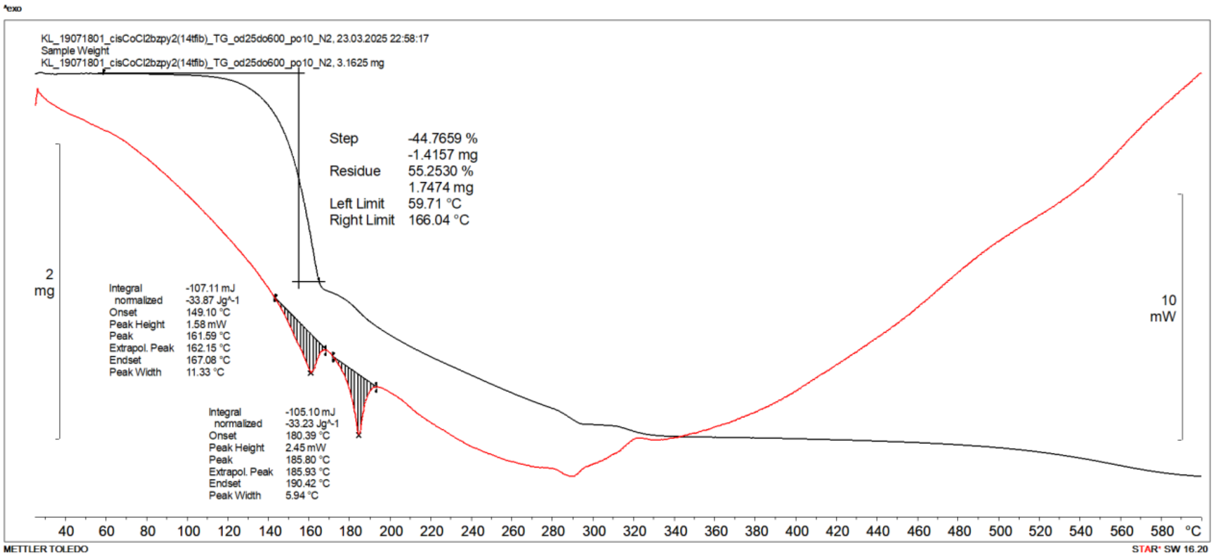

Figure S17. TGA (black curve) and DSC (red curve) thermograms for (*cis*-$CoCl_2$**bzpy**$_2$)(**14tfib**).

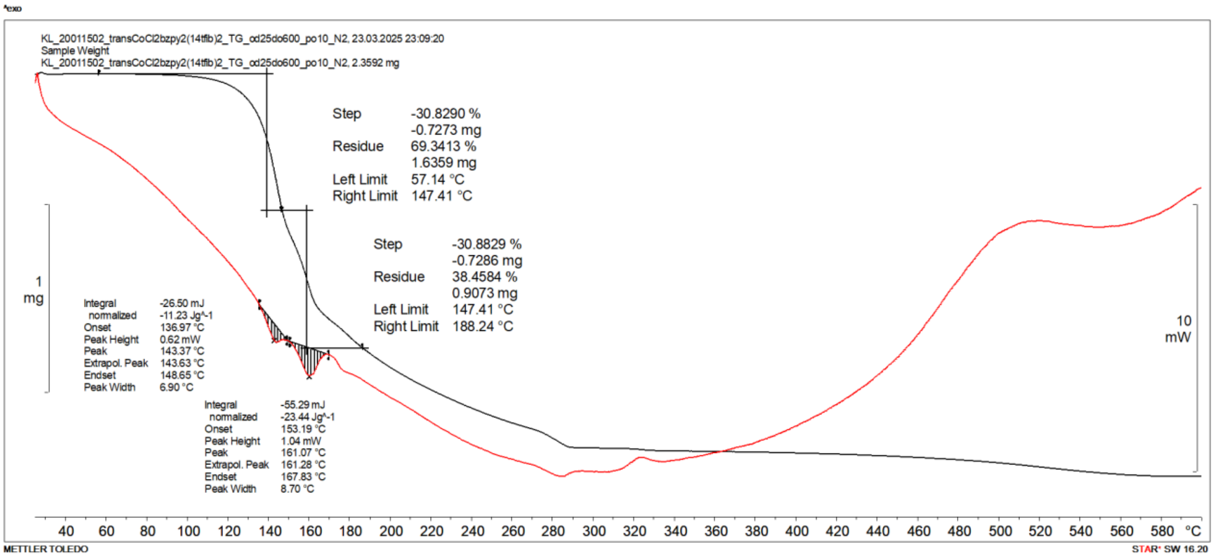

Figure S18. TGA (black curve) and DSC (red curve) thermograms for (*trans*-$CoCl_2$**bzpy**$_2$)(**14tfib**)$_2$.

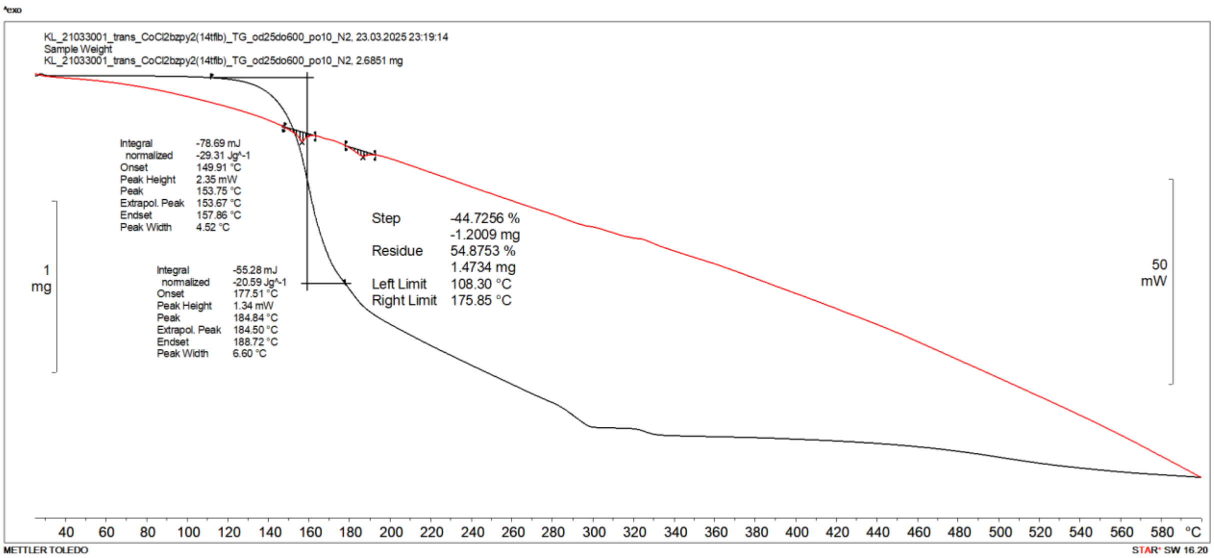

Figure S19. TGA (black curve) and DSC (red curve) thermograms for (*trans*-$CoCl_2$**bzpy**$_2$)(**14tfib**).

8. Theoretical calculations

Molecular DFT calculations on an isolated molecule of $CoCl_2$**bzpy**$_2$ in its *cis* and *trans* forms was performed using Gaussian 16 version B.01.[40] The structures were geometry-optimized under PBE/6-311G(d,p) level of theory.[41] Each molecule was optimized both in the high spin (S=3/2) and low spin (S=1/2) configuration. Geometries were deemed converged upon satisfying the following criteria: maximum force 4.5x10^{-4} a. u.; RMS force 3.0x10^{-4} a. u.; maximum displacement 1.8x10^{-3} a. u.; RMS displacement 1.2x10^{-3} a. u.

Periodic DFT calculations were performed with CASTEP 19 plane wave DFT code.[42] The experimental crystal structures in CIF format were converted into CASTEP input files using the program cif2cell.[54] The crystal structures were geometry-optimized using the PBE functional[40] combined with a many body dispersion correction (MBD*).[43] Calculations were performed in a spin-polarized mode in order to account for the spin magnetic moment of th Co^{2+} cations. During the optimization both unit cell and atom coordinates were relaxed, while maintaining the constraints imposed by space group symmetry. The plane wave basis set was truncated at 800 eV cutoff, and ultrasoft pseudopotentials were used for the core regions of electron density. The 1st electronic Brillouin zone was sampled with a 0.05 Å^{-1} Monkhorst–Pack k-point grid. The structure optimization was deemed complete upon satisfyng the following convergence criteria: maximum energy change of 2x10-5 eV/atom, maximum force on atom of 0.05 eV Å^{-1}, maximum atom dispalacement of 10^{-3} Å and maximum component of the stress tensor of 0.1 GPa.

Table S5. Energy values of *cis*- and *trans*-$CoCl_2$**bzpy**$_2$ for isolated molecules.

	High-spin state	Low-spin state
E (*cis*-$CoCl_2$**bzpy**$_2$)/kJ mol^{-1}	-9154717.206	-9154745.211
E (*trans*-$CoCl_2$**bzpy**$_2$)/kJ mol^{-1}	-9154709.004	-9154757.948

Table S6. Periodic DFT electronic energies of *cis*-$CoCl_2$**bzpy**$_2$, **14tfib** and cocrystals.

Structure	Electronic energy per primitive unit cell / eV	Electronic energy per formula unit/ eV
cis-$CoCl_2$**bzpy**$_2$	-29810.140	-7452.535
**14tfib**	-10365.148	-5182.574
(*cis*-$CoCl_2$**bzpy**$_2$)(**14tfib**)	-25270.287	-12635.143
(*cis*-$CoCl_2$**bzpy**$_2$)(**14tfib**)$_2$	-71271.117	-17817.779
(*trans*-$CoCl_2$**bzpy**$_2$)(**14tfib**)	-12635.870	-12635.870
(*trans*-$CoCl_2$**bzpy**$_2$)(**14tfib**)$_2$	-17818.616	-17818.616

References

[45] T. Degen, M. Sadki, E. Bron, U. König, G. Nénert, *Powder Diffr.* **2014**, *29*, S13–S18.

[46] CrysAlis CCD V171.34, Oxford Diffraction, **2003**, Oxford Diffraction Ltd., Abingdon, Oxfordshire, UK.

[47] CrysAlis RED V171.34, Oxford Diffraction, **2003**, Oxford Diffraction Ltd., Abingdon, Oxfordshire, UK.

[48] G. M. Sheldrick, "Crystal structure refinement with SHELXL" *Acta Crystallogr.* **2015**, *A71*, 3.

[49] G. M. Sheldrick, "A short history of SHELX" *Acta Crystallogr.* **2008**, *A64*, 112.

[50] L. J. Farrugia, "WinGX suite for small-molecule single-crystal crystallography" *J. Appl. Crystallogr.* **1999**, *32*, 837.

[51] C. F. Macrae, I. J. Bruno, J. A. Chisholm, P. R. Edgington, P. McCabe, E. Pidcock, L. Rodriguez-Monge, R. Taylor, J. Van De Streek, P. A. Wood, "Mercury CSD 2.0 – new features for the visualization and investigation of crystal structures" *J. Appl. Crystallogr.* **2008**, *41*, 466.

[52] Topas V5, Bruker AXS, **2014**.

[53] STARe Software v.15.00., Mettler-Toledo GmbH, **2016**.

[54] T. Björkman, "CIF2Cell: Generating geometries for electronic structure programs" *Comput. Phys. Commun.* **2011**, *182*, 1183.
